# Combining decitabine with radiotherapy to enhance nasopharyngeal carcinoma radiosensitivity via the TFAP2C-OTUD1-SLC25A11 axis

**DOI:** 10.1038/s41419-025-07858-9

**Published:** 2025-07-15

**Authors:** Haixia Zhang, Siyang Liu, Dan Wang, Yaqi Liao, Shizhen Li, Jing He, Jie Shen, Lu Yan, Tengfei Xiao, Wangning Gu, Hongmin Yang, Hui Wang, Minghua Yang, Pan Chen

**Affiliations:** 1 The Affiliated Cancer Hospital of Xiangya School of Medicine, Central South University/Hunan Cancer Hospital, Changsha, China; 2 Department of Pediatrics, Third Xiangya Hospital, Central South University, Changsha, China

**Keywords:** Cancer therapeutic resistance, Cancer models

## Abstract

Nasopharyngeal carcinoma (NPC) is a common malignancy in certain geographic regions, with radiotherapy serving as the primary treatment. Recent research shows that epigenetics and deubiquitinases (DUBs) are crucial in NPC progression and treatment response. However, the emergence of radioresistance in NPC cells presents a significant challenge, often resulting in treatment failure. This study focuses on understanding the role of OTUD1 and methylation in NPC radiosensitivity and their mechanisms. In this study, OTUD1 and TFAP2C expression were significantly reduced in radioresistant NPC cell lines, likely due to the high methylation of TFAP2C. OTUD1 is significantly downregulated in radioresistant NPC, and its low expression is associated with enhanced radioresistance both in vitro and in vivo. Mechanistically, OTUD1 enhances NPC radiosensitivity by deubiquitinating and stabilizing SLC25A11, leading to increased Reactive oxygen species (ROS) and apoptosis. Clinically, low OTUD1 and SLC25A11 expression is associated with poor radiotherapy response and survival outcomes. Furthermore, we demonstrate that combining the methylation inhibitor Decitabine (DAC) with radiotherapy significantly improves treatment efficacy by overcoming radioresistance. These findings provide insights into NPC radioresistance and suggest that using DAC in combination with radiotherapy to target the TFAP2C-OTUD1-SLC25A11 axis could be a promising strategy to overcome radioresistance.

## Introduction

NPC is a malignant tumor originating from the nasopharynx and is characterized by unique geographic distribution and ethnic predisposition [1, 2]. Radiotherapy is the primary treatment for NPC, particularly in locally advanced and recurrent cases. Recent advancements in tumor localization, radiotherapy, and combination therapies have significantly improved patient survival [3–6]. However, some patients develop radioresistance, resulting in poor treatment outcomes. Therefore, understanding the mechanisms of radioresistance in NPC and identifying methods to restore radiosensitivity are crucial for enhancing therapeutic efficacy and patient survival.

DUBs are enzymes that regulate protein stability and activity by removing ubiquitin, playing key roles in cell cycle control, tumor progression, and treatment sensitivity [7–11]. The OTU family is a subgroup of DUBs characterized by a specific OTU (ovarian tumor) domain, which is involved in the removal of ubiquitin from target proteins. As a member of this family, OTUD1 has shown tumor-suppressive effects in cancers such as lung and liver cancer [12–15], but its role in NPC remains unexplored, warranting further research.

SLC25A11 is a mitochondrial carrier protein that transports α-ketoglutarate (α-KG) from the mitochondria to the cytoplasm, playing a key role in maintaining cellular redox balance [16]. Research indicates that SLC25A11, a mitochondrial Glutathione (GSH) transporter, regulates ROS levels to influence cellular metabolism and tumor proliferation, contributing significantly to oxidative stress regulation in various cancers [17–19]. However, its role in NPC and impact on radiation sensitivity remain unclear. While SLC25A11 is closely tied to oxidative stress, its influence on NPC cell response, particularly during radiotherapy, is still not well understood. Therefore, further investigation into the role of SLC25A11 in NPC radiotherapy may provide new insights and strategies for enhancing treatment efficacy.

DNA methylation is crucial in tumor initiation and progression, often leading to the silencing of tumor suppressor genes. Abnormal methylation is linked to tumor growth, invasion, and treatment resistance [11, 20–24]. The demethylation inhibitor DAC has been studied in cancers like AML, where it induces apoptosis, and in lung and breast cancers, where it enhances radiotherapy efficacy [25–31]. However, the mechanism of combining DAC with radiotherapy in NPC remains unstudied and requires further exploration.

TFAP2C is a key transcription factor involved in regulating various cancers [32–35]. As a core regulator in breast cancer, it promotes cell growth and malignant proliferation by activating genes like HER2, ER, and Cyclin D1 [33, 36–39]. TFAP2C is also associated with the malignant phenotypes of ovarian and colorectal cancers by regulating genes linked to the cell cycle, apoptosis, and EMT, enhancing tumor growth and invasion [34, 35]. Currently, the role of TFAP2C in regulating OTUD1 in NPC is unreported, indicating a direction for future research.

Here, we found that OTUD1 promotes apoptosis under radiotherapy in NPC, and its low expression correlates with poorer prognosis and radiotherapy resistance. We demonstrated that OTUD1 deubiquitinates and stabilizes mitochondrial GSH transporter SLC25A11, increasing ROS to enhance radiosensitivity. Additionally, we discovered that radiotherapy resistance in NPC is likely linked to hypermethylation of the transcription factor TFAP2C. Further experiments using methylation inhibitors combined with radiotherapy showed improved treatment sensitivity. These insights into the role of DUBs and methylation inhibitors in NPC radioresistance suggest strategies for enhancing radiosensitivity by targeting specific DUBs and their interacting proteins.

## Materials and methods

### Cell culture and establishment of radioresistant cell lines

NPC cell lines, CNE2 and HONE1, were obtained from the Central Experiment Laboratory of Xiangya Medical School, Central South University. Exponentially growing CNE2 and HONE1 cells were seeded at a density of 1 × 10^5^ cells per T25 flask and initially exposed to 2 Gy of IR. Following irradiation, cells were cultured in RPMI-1640 medium (Gibco, USA) supplemented with 10% fetal bovine serum (FBS, Gibco, USA), 100 IU/mL penicillin, and 100 IU/mL streptomycin. Cells were passaged twice before receiving another 2 Gy of IR. This procedure was repeated with increasing doses of IR (4, 6, 8, and 10 Gy), with a total accumulated dose of 60 Gy over 6 months. Surviving CNE2 and HONE1 cells were designated as radioresistant (CNE2-RR and HONE1-RR, respectively). Control cells, treated similarly without IR, were designated as CNE2-RS and HONE1-RS. All experiments utilized cells within 5–10 passages after the final IR exposure.

CNE2-RR, CNE2-RS, HONE1-RR, and HONE1-RS cells were cultured in RPMI-1640 medium with 10% FBS, 100 IU/mL penicillin, and 100 IU/mL streptomycin at 37 °C in a 5% CO₂ humidified atmosphere. All experiments used exponentially growing cells.

### Drug treatments

Cells were treated with DAC, cycloheximide (CHX), and MG132, as well as cell death inhibitors, including VAD (apoptosis inhibitor), Nec1 (necroptosis inhibitor), Necrostatin-1 (Necr, necroptosis inhibitor), Fer-1 (ferroptosis inhibitor), Lip-1 (ferroptosis inhibitor), 7559 (LDC7559, pyroptosis inhibitor), and CQ (chloroquine, autophagy inhibitor). For DAC (Sigma) treatment, cells were cultured for 24 h before being exposed to 1–10 µM DAC, with medium replacement every 24 h for 72 h, following 6 Gy IR or no IR. For MG132 (Sigma) treatment, transfected cells were incubated with 10 µM MG132 for 6 h. For CHX (Sigma) treatment, transfected cells were incubated with 100 µg/mL CHX for different durations (0, 2, 4, 8, 12, and 16 h). For VAD treatment, transfected cells were incubated with 25 µM VAD for 24 h. For Nec1 treatment, transfected cells were incubated with 2 µM Nec1 for 24 h. For Necrostatin-1 treatment, transfected cells were incubated with 5 µM Necr for 24 h. For Fer-1 treatment, transfected cells were incubated with 2 µM Fer-1 for 24 h. For Lip-1 treatment, transfected cells were incubated with 5 µM Lip-1 for 24 h. For 7559 treatment, transfected cells were incubated with 3 µM 7559 for 24 h. For CQ treatment, transfected cells were incubated with 5 µM CQ for 24 h.

### Plasmid construction and transfection

The coding regions of OTUD1, SLC25A11, and TFAP2C were tagged with FLAG, HA, and FLAG, respectively, and cloned into the pcDNA3.1 vector to create overexpression plasmids, pcDNA3.1-OTUD1-FLAG, pcDNA3.1-SLC25A11-HA, and pcDNA3.1-TFAP2C-FLAG. The coding sequences were amplified by PCR and inserted into the pcDNA3.1 using standard molecular cloning techniques, including restriction enzyme digestion and ligation.

For transient transfection, cells were transfected with overexpression plasmids using Lipofectamine 3000 (Invitrogen) according to the manufacturer’s protocol. Briefly, the cells were seeded in 6-well plates and allowed to reach 70-80% confluency. The transfection mixture was prepared by combining the plasmid DNA with Lipofectamine 3000 and P3000 reagent in Opti-MEM medium. This mixture was then added to the cells. The cells were incubated with the transfection complexes for 24–48 h before harvest.

For stable transfection, lentiviruses containing all relevant genes were obtained from Genechem Co., Ltd Lentiviral transfection was performed using the HitransG P reagent. Infected cells were selected with puromycin (0.5–1 μg/ml) 48 h post-infection. Stable cell lines were maintained in a puromycin-containing medium and harvested for RT-qPCR and western blotting to assess target gene expression.

### RT-qPCR assay

Total cellular RNA was extracted using an RNAfast200 Kit (Fastagen). cDNA was synthesized using the Evo M-MLV RT Mix Kit (Accurate Biology). qRT-PCR was performed using the SYBR® Green Premix Pro Taq HS qPCR Tracking Kit (Accurate Biology), following the manufacturer’s instructions. All primers used for qRT-PCR are listed in Supplementary Table S1.

### Western blot assay

Cells were collected by centrifugation and lysed on ice using RIPA buffer supplemented with PMSF. After high-speed centrifugation, the supernatant was collected, and protein concentrations were measured using the Pierce™ BCA Protein Assay Kit (Thermofisher). Proteins were separated using polyacrylamide gel electrophoresis and transferred onto PVDF membranes. Membranes were blocked with 5% skim milk for 1 h at room temperature, then incubated overnight with primary antibodies at 4 °C. The next day, the membranes were incubated with HRP-conjugated goat anti-rabbit/mouse IgG (H+L) secondary antibodies for 1 h at room temperature. All antibodies used are listed in Supplementary Table S2. Protein signals were detected using the ChampChemi imaging system (SINSAGE).

### Immunofluorescence and confocal microscopy

Adherent cells were fixed in 4% paraformaldehyde for 15 min and permeabilized with 0.5% Triton X-100 for 10 min. Blocking was done with 1% BSA in PBS for 1 h at room temperature. Primary antibodies (listed in Supplementary Table S2) were applied and incubated overnight at 4 °C. The next day, cells were washed and incubated with secondary antibodies for 1 h at room temperature. Nuclei were stained with 4′,6-diamidino-2-phenylindole (DAPI, Sigma) for 5 min. Coverslips were mounted to prevent quenching, and fluorescence images were obtained using a confocal scanning microscope (LSM880 with Fast Airyscan, ZEISS).

### Mass spectrometry (MS) and co-immunoprecipitation (co-IP) assays

For the co-IP assay, cells were lysed on ice using IP lysis buffer and sonicated. The total protein lysate was incubated overnight at 4 °C with 3 μg of specific antibodies for IP. The immune complexes were combined with Pierce™ Protein A/G Magnetic Beads (Thermo Scientific) and washed with IP wash buffer. The isolated immune complexes were separated by SDS-PAGE and visualized by Coomassie blue staining. MS was performed using Baipu Biotechnology (China). Target proteins from the co-IP were identified using western blot analysis. The antibodies used are listed in Supplementary Table S2.

### IR treatment

Treated cells were exposed to in vitro radiotherapy, with the radiation dose set at 6 Gy unless otherwise specified. In mouse experiments, radiation therapy at 12 Gy was administered once the tumors reached an appropriate size (7–14 days post-implantation).

### Cell viability assay

Cells were seeded into 96-well plates at a density of 1500 cells per well. To assess treatment effects on cell growth and viability, 10 μl of Cell Counting Kit-8 reagent was added on days 0, 1, 2, 3, and 4. After a 2-h incubation at 37 °C, absorbance was measured at 560 nm using a microplate reader, with results expressed as OD values.

### Clonogenic assay

Cells were seeded into 6-well plates at densities of 1000–8000 cells per well and allowed to adhere. After adherence, the cells were exposed to irradiation doses ranging from 0 to 8 Gy. After 10–14 days, when colonies formed, the plates were rinsed with PBS, fixed with methanol, and stained with crystal violet. Colonies containing more than 50 cells were counted.

### Flow cytometry analysis of cell apoptosis

The apoptosis rate of each sample was evaluated using the Annexin V-FITC/PI Apoptosis Detection Kit. Cells were harvested 48 h post-irradiation, washed twice with PBS, and resuspended in 500 μl of binding buffer. Subsequently, 5 μl of Annexin V-FITC and 5 μl of PI fluorescent dyes were added. The apoptotic cells were detected by CytoFLEX flow 367 cytometers. In this assay, FITC-/PI- cells were considered viable, FITC+/PI− cells as early apoptotic, and FITC+/PI+ cells as late apoptotic or dead.

### Chromatin immunoprecipitation (ChIP) and DNA gel electrophoresis

ChIP was performed by crosslinking DNA-protein complexes in harvested cells with formaldehyde, followed by quenching with glycine. Cells were lysed, and chromatin was fragmented via sonication. The chromatin was incubated overnight with specific antibodies at 4 °C, followed by the addition of protein A/G magnetic beads, which were incubated for 2 h. The beads were washed, and the DNA-protein complexes were eluted and de-crosslinked by heating. DNA was purified using a PCR purification kit and amplified with the top five primers from the ChIP-seq databases. For DNA gel electrophoresis, a 1.5% agarose gel was prepared, and DNA samples mixed with loading dye were run at 80 V. The DNA bands were visualized under UV light and imaged using a gel documentation system.

### Methylation-specific PCR (MSP)

Genomic DNA was extracted from cells using the TIANamp Genomic DNA Kit (Tiangen). The DNA was bisulfite-converted using the DNA Bisulfite Conversion Kit (Tiangen) according to the manufacturer’s protocol. After bisulfite modification, the DNA was amplified using an MSP Kit (Tiangen). The PCR products were resolved on 2.5% agarose gels and visualized with ethidium bromide staining. The primer sequences used for MSP are listed in Supplementary Table S1.

### Bisulfite sequencing PCR

Genomic DNA was extracted from cells using the TIANamp Genomic DNA Kit (Tiangen) and treated with a DNA Bisulfite Conversion Kit (Tiangen) following the manufacturer’s instructions. Aliquots of bisulfite-treated DNA were subjected to 45 PCR amplification cycles. PCR primers were designed using the CpG island search engine MethPrimer for the regions with the highest methylation from MSP (sequences in Supplementary Table S1). The final PCR products were cloned using the pMD19-T Vector Cloning Kit (Takara) and sequenced on an ABI Prism 3730XL DNA Sequencer (Applied Biosystems, USA). Data were obtained from at least five valid samples for each promoter region.

### Comet assay

The cells were collected 48 h post-irradiation (6 Gy). The detection was performed using the Beyotime Comet Assay Kit (Beyotime Biotechnology) and staining with propidium iodide. Quantitative analysis of comet tail moments was conducted using the CaspLab-Comet Assay Software, with 20 cells scored per sample.

### Detection of ROS

To detect cellular ROS, cells were collected 48 h post-radiotherapy. CellROX Green and CellROX Deep Red dyes were used at a concentration of 5 µM. Cultured cells were collected, centrifuged, and stained in 500 µL of pre-warmed PBS containing the dye for 30 min at 37 °C, protected from light. After staining, the cells were washed with 1X pre-warmed PBS, transferred to capped flow tubes, and kept on ice during acquisition.

### GSH detection

The concentration of GSH in the samples was determined using a Beyotime GSH Assay Kit. Reagents were added to initiate the reaction according to the manufacturer’s instructions. The absorbance was measured at 412 nm using a microplate spectrophotometer. The GSH concentration in each sample was calculated using a standard curve.

### Mouse models

Female BALB/c nude mice (3-4 weeks old) were acquired from Hunan Silaike Jingda Experimental Animal Co., Ltd The mice were randomly assigned to one of the four groups and injected subcutaneously with 2 × 10^6^ CNE2-RR cells or CNE2-RR cells overexpressing OTUD1. When tumor volume reached 50–100 mm^3^, the radiotherapy group received localized irradiation totaling 12 Gy in two sessions with only the tumor area irradiated and the rest shielded using lead plates over 5 mm thick. Tumor dimensions were measured every 3 days, and the volume was calculated using the formula: volume = 1/2 × length × width^2^. Tumors were excised from the euthanized mice, weighed, and embedded in paraffin for immunohistochemistry (IHC). Animal experiments were conducted with approval from the Animal Welfare and Ethics Committee of Hunan Cancer Hospital (approval no. 2022177).

### Clinical specimens, immunohistochemistry, and histological staining

This study was approved by the Animal Welfare and Ethics Committee of Hunan Cancer Hospital (approval no. KYJJ-2022-304). Paraffin-embedded and fresh tumor tissues were obtained from patients with non-metastatic NPC who received radical radiotherapy or chemoradiotherapy. Radiosensitive cases showed complete regression or no recurrence, while radioresistant cases exhibited incomplete regression, residual disease beyond 6 weeks, or recurrence. Fresh samples were snap-frozen in liquid nitrogen and stored at −80 °C.

Paraffin-embedded sections were used for IHC and HE staining. For IHC, sections were deparaffinized, rehydrated, and treated with hydrogen peroxide to block endogenous peroxidase activity. Sections were then blocked with goat serum and incubated with primary antibodies overnight at 4 °C. After washing, HRP-conjugated rabbit/mouse secondary antibodies were applied. Diaminobenzidine (DAB) was used for chromogenic detection, and hematoxylin was used for counterstaining. For HE staining, sections were stained with hematoxylin to visualize nuclei and eosin for cytoplasmic structures. Images were captured using the AxioVision Rel.4.6 system (Carl Zeiss).

IHC results were evaluated by two experienced pathologists using the Immunoreactive Score (IRS) system. Staining intensity was graded as follows: 0 (negative), 1 (weak), 2 (moderate), and 3 (strong). The percentage of positively stained tumor cells was scored as: 1 (<10%), 2 (10–35%), 3 (35–70%), and 4 (>70%). The final IRS score was calculated as the product of these two parameters. Antibodies used for IHC analysis are listed in Supplementary Table S2.

### Statistical analysis

SPSS version 19.0 (IBM Corp., Armonk, NY, USA) and GraphPad Prism software were applied in the statistical analysis. The statistical tests for data analysis included the *χ*^2^ test, two-tailed Student’s *t* test, log-rank test, Spearman-rank correlation test, and one-way or two-way ANOVA. Data are expressed as the mean ± SD. Sig nificant difference was defined by *P ≤ 0.05, **P ≤ 0.01, ***P ≤ 0.001, and ****P ≤ 0.0001

## Results

### OTUD1 promotes ROS accumulation and radiosensitivity in NPC cells

To address the radioresistance often seen in NPC, we established radioresistant cell lines, CNE2-RR and HONE1-RR, by subjecting parental NPC cell lines to multiple rounds of low-dose irradiation. These lines exhibited significantly enhanced radioresistance compared to the parental cells (Supplementary Fig. 1).

**Fig. 1:**
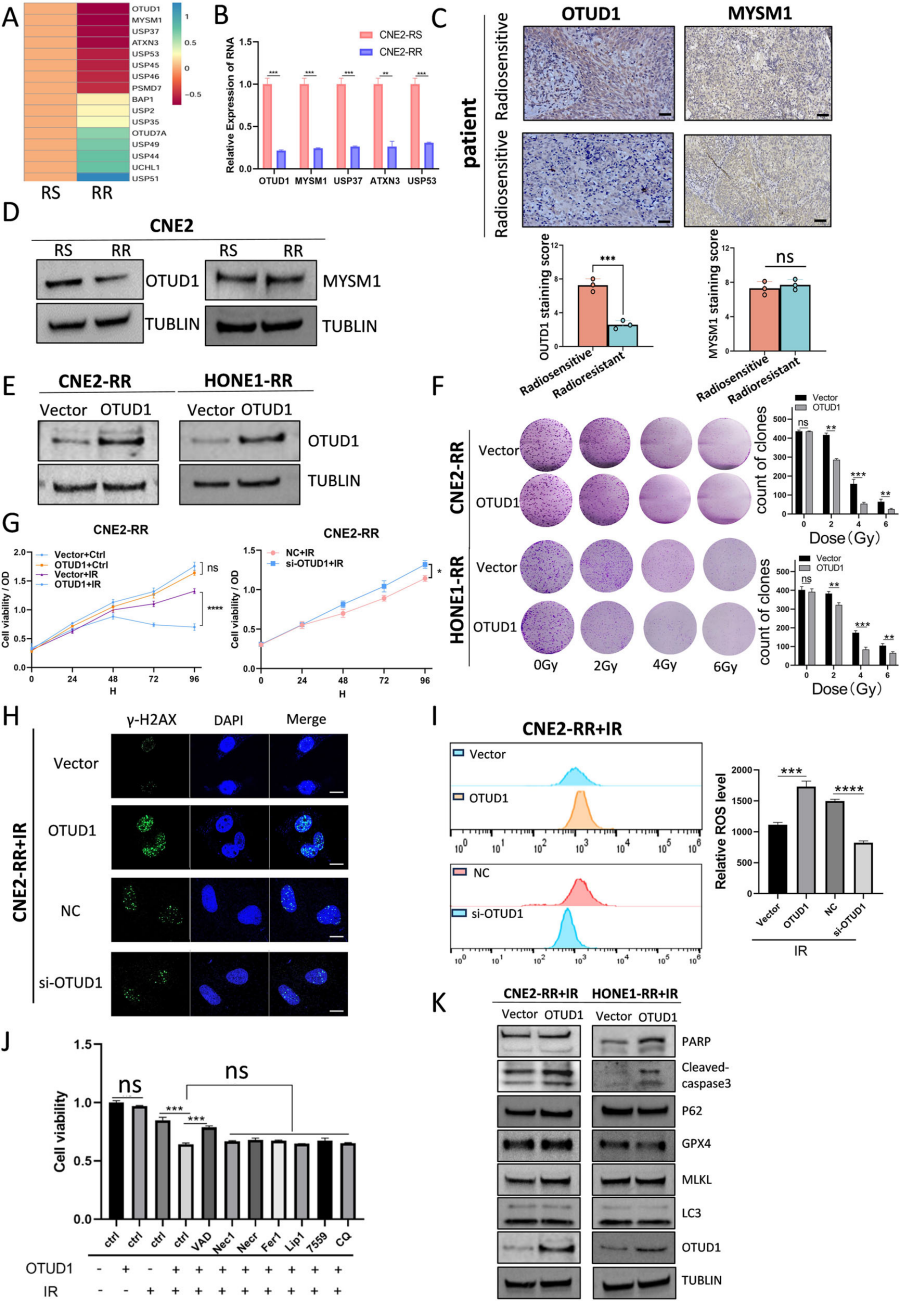
OTUD1 promotes ROS accumulation and radiosensitivity in NPC cells. **A** qPCR screening of 90 deubiquitinases in radioresistant and radiosensitive NPC cell lines. **B** qPCR validation of OTUD1, MYSM1, and USP51 expression. **C**, **D** IHC and WB analyses of OTUD1 and MYSM1 expression in NPC patient tissues and cell lines. **E** WB analysis confirming OTUD1 overexpression in NPC cell lines. **F**, **G** Clonogenic and proliferation assays showing OTUD1 overexpression impairs NPC survival post-irradiation. **H** Immunofluorescence staining of γ-H2AX to assess DNA damage in OTUD1-overexpressing cells post-irradiation. Scale bars, 10 μm. **I** ROS measurement shows that OTUD1 overexpression increases ROS production under irradiation. **J** CCK-8 assay demonstrating apoptosis-dependent reduction in cell viability upon OTUD1 overexpression post-irradiation. **K** WB analysis showing OTUD1 enhances apoptosis (PARP, Cleaved-Caspase3) but not autophagy, ferroptosis, or necroptosis post-irradiation. Data are presented as means ± S.D. with *P* values determined using the two-tailed Student’s *t*-test; *n* = 3 independent experiments.

To investigate the role of deubiquitinases in radioresistance, we compiled 90 previously reported deubiquitinating enzymes and analyzed their expression in radioresistant and radiosensitive NPC cell lines using qPCR. This screening identified several differentially expressed deubiquitinases, with OTUD1 and MYSM1 showing the most significant downregulation in radioresistant cells, while USP51 was the most upregulated (Fig. 1A, B and Supplementary Fig. 2A). However, despite the marked differential expression of USP51, its overall expression level remained exceedingly low, which may limit its biological significance in radioresistance (Supplementary Fig. 2B). Therefore, we focused on OTUD1 and MYSM1 to further investigate their potential roles and underlying mechanisms in radioresistance. To validate our findings, we performed IHC to assess OTUD1 and MYSM1 expression in tissue samples from radiosensitive and radioresistant NPC patients, and WB to analyze their expression in NPC cell lines. OTUD1 was significantly lower in radioresistant patients, consistent with qPCR results, while MYSM1 showed no significant difference (Fig. 1C, D and Supplementary Fig. 2C). Based on these findings, we selected OTUD1 for further investigation in NPC radioresistance.

To further investigate the role of OTUD1 in NPC radioresistance, we established radioresistant cell lines with stable overexpression or transient knockdown of OTUD1 (Fig. 1E and Supplementary Fig. 3A). Overexpression of OTUD1 had minimal effects under non-irradiated conditions but significantly impaired colony formation and cell proliferation in NPC cells following irradiation (Fig. 1F, G and Supplementary Fig. 2D). In contrast, knockdown of OTUD1 promoted cell survival and proliferation under irradiation (Supplementary Fig. 3B–D) and attenuated DNA damage effects (Supplementary Fig. 3E). Meanwhile, overexpression of OTUD1 increased DNA damage and further enhanced radiosensitivity (Fig. 1H and Supplementary Fig. 2E).

Previous studies linked low ROS levels to radioresistance in cancer stem cells [40–42]. In line with these findings, our analysis showed that radioresistant cells consistently exhibited reduced ROS levels, even under irradiation (Supplementary Fig. 1E). Notably, OTUD1 was identified as a key regulator of this response: its overexpression elevated ROS levels and increased radiosensitivity, whereas its knockdown led to diminished ROS production and enhanced radioresistance (Fig. 1I and Supplementary Fig. 3F).

To further elucidate how OTUD1 enhances radiosensitivity, we compared various cell death inhibitors. Only the apoptosis inhibitor effectively reversed OTUD1-induced cell death, indicating that apoptosis plays a key role in its radiosensitizing effects (Fig. 1J). Consistently, Western blot analysis confirmed these findings. Under radiation, OTUD1 overexpression had no significant effect on autophagy (P62, LC3), ferroptosis (GPX4), or necroptosis (p-MLKL) proteins but significantly increased apoptosis-related proteins PARP and Cleaved-Caspase3, further supporting the conclusion that OTUD1 enhances radiosensitivity primarily by promoting apoptosis rather than other forms of cell death (Fig. 1K). Flow cytometry showed increased apoptosis rates in radioresistant cells overexpressing OTUD1 under irradiation (Supplementary Fig. 2F). Knocking down OTUD1 inhibited apoptosis under irradiation (Supplementary Fig. 3G, H).

These results suggest that OTUD1 sensitizes radioresistant NPC cells to irradiation by increasing ROS levels and inducing apoptosis, highlighting its critical role in enhancing radiosensitivity.

### OTUD1 stabilizes SLC25A11 by reducing its ubiquitination

**Fig. 2:**
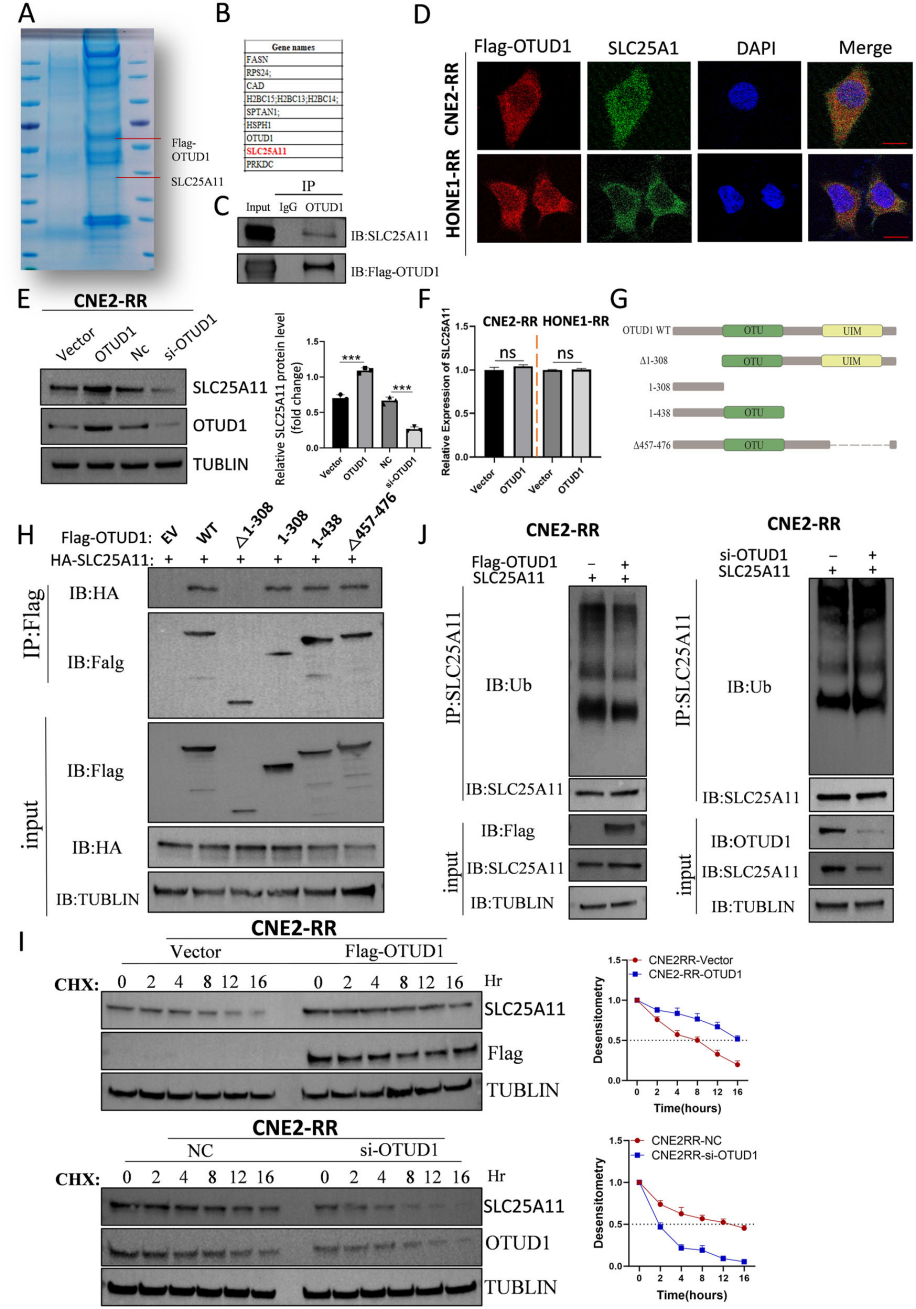
OTUD1 stabilizes SLC25A11 by reducing its ubiquitination. **A**, **B** Mass spectrometry (MS) identified SLC25A11 as an OTUD1-interacting protein. **C**, **D** Co-immunoprecipitation (Co-IP) and immunofluorescence assays confirmed the exogenous interaction between OTUD1 and SLC25A11. Scale bars, 5 μm. **E** WB analysis showing that overexpression of OTUD1 enhances SLC25A11 expression, while silencing OTUD1 inhibits SLC25A11 expression in CNE2-RR cells. **F** Overexpression of *OTUD1* without significantly affecting SLC25A11 mRNA levels. **G**, **H** Truncation analysis revealed that residues 1-308 of OTUD1 are essential for the interaction with SLC25A11, as evidenced by IP analysis. **I** Half-life analysis of endogenous SLC25A11 in CNE2-RR cell, performed by overexpressing or silencing OTUD1, followed by protein stability assessment over time. **J** Ubiquitination assay to assess the polyubiquitination of SLC25A11 in CNE2-RR cell, with OTUD1 overexpression or silencing, using immunoprecipitation and WB analysis. Data are presented as means ± S.D. with *P* values determined using the two-tailed Student’s *t*-test; *n* = 3 independent experiments.

To investigate the mechanism of OTUD1 as a deubiquitinase, we performed IP coupled with MS analysis to identify OTUD1-interacting proteins. Previous studies have demonstrated that ROS play a crucial role in the development of radioresistance in NPC, highlighting the importance of redox regulation in this process. Among the top ten OTUD1-interacting proteins identified in our MS analysis, we found SLC25A11, a key mitochondrial GSH transporter (Fig. 2A, B), suggesting its potential role in OTUD1-mediated radiosensitivity regulation. Co-IP and immunofluorescence assays confirmed the exogenous interaction between OTUD1 and SLC25A11 (Fig. 2C, D). Additionally, SLC25A11 expression was significantly downregulated in radioresistant NPC cells, consistent with the expression pattern of OTUD1 (Supplementary Fig. 4A). To further investigate the effect of OTUD1 on SLC25A11 expression, we performed experiments by overexpressing or knocking down OTUD1. The results showed that overexpression of OTUD1 significantly increased SLC25A11 protein levels by promoting its deubiquitination, while knockdown of OTUD1 led to a decrease in SLC25A11 protein levels (Fig. 2E, F). Importantly, these changes were observed only at the protein level of SLC25A11, with no significant alterations at the RNA level (Supplementary Fig. 4B). Thus, we hypothesized that OTUD1 deubiquitinates and stabilizes SLC25A11.

We performed a truncation analysis to identify the regions responsible for mediating the OTUD1-SLC25A11 interaction (Fig. 2G). IP analysis showed that after truncating the OUT and UIM domains, OTUD1 still bound to SLC25A11. However, mutating residues 1-308 of OTUD1 completely abolished the interaction between the two proteins, highlighting the critical role of residues 1-308 in this interaction (Fig. 2H). Overexpression of OTUD1 significantly prolonged the half-life of endogenous SLC25A11, while silencing OTUD1 shortened it (Fig. 2I and Supplementary Fig. 4C). Furthermore, OTUD1 overexpression reduced the ubiquitination of SLC25A11, while silencing OTUD1 increased its ubiquitination (Fig. 2J and Supplementary Fig. 4D). Thus, OTUD1 promotes the deubiquitination and stabilization of SLC25A11 in NPC.

### SLC25A11 enhances radiosensitivity in NPC cells

**Fig. 3:**
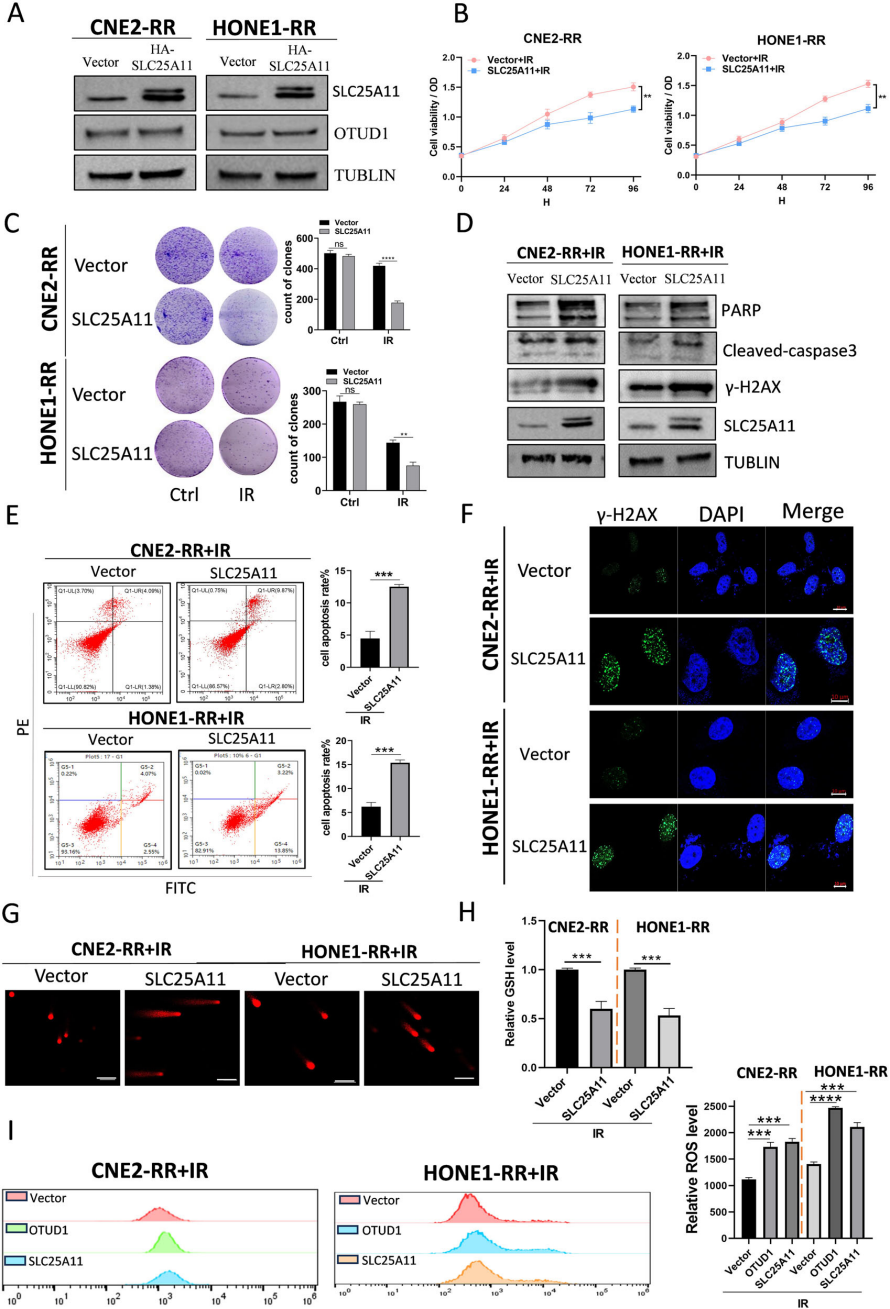
SLC25A11 Enhances Radiosensitivity in NPC Cells. **A** WB analysis to validate the overexpression of SLC25A11 in CNE2-RR and HONE1-RR cell lines, while also examining the impact of SLC25A11 overexpression on OTUD1 expression levels. **B**, **C** Colony formation and proliferation assays showing that SLC25A11 overexpression enhances radiosensitivity and inhibits cell proliferation post-irradiation. **D**, **E** Apoptosis assays demonstrate increased apoptosis in SLC25A11-overexpressing cells after irradiation. **F**, **G** The Immunofluorescence analysis for γ-H2AX and comet assays indicate higher levels of DNA damage in SLC25A11-overexpressing cells under irradiation conditions. Scale bars, 10 μm and 100 μm. **H**, **I** Analysis of intracellular GSH and ROS levels shows that SLC25A11 overexpression decreases GSH levels and increases ROS production following irradiation. **D** Data are presented as means ± S.D. with *P* values determined using the two-tailed Student’s *t*-test; *n* = 3 independent experiments.

To examine the impact of SLC25A11 on the radiosensitivity of radioresistant cells, we established stable overexpression cell lines for CNE2-RR and HONE1-RR, and found that overexpression of SLC25A11 did not affect the expression of OTUD1 (Fig. 3A). Under irradiation, overexpression of SLC25A11 significantly enhanced radiosensitivity, inhibited cell proliferation (Fig. 3B, C), promoted apoptosis (Fig. 3D, E), and increased DNA damage (Fig. 3F, G).

We assessed the changes in GSH and ROS levels under irradiation. Consistent with our hypothesis, SLC25A11 overexpression led to decreased intracellular GSH levels and increased ROS production (Fig. 3H, I). These results demonstrate that SLC25A11 enhances the radiosensitivity of radioresistant NPC cells. Overexpression of SLC25A11 promotes apoptosis and DNA damage while modulating intracellular GSH and ROS levels and contributing to the increased radiosensitivity observed in these cells.

### OTUD1 enhances radiosensitivity in radioresistant NPC cells through SLC25A11 stabilization

**Fig. 4:**
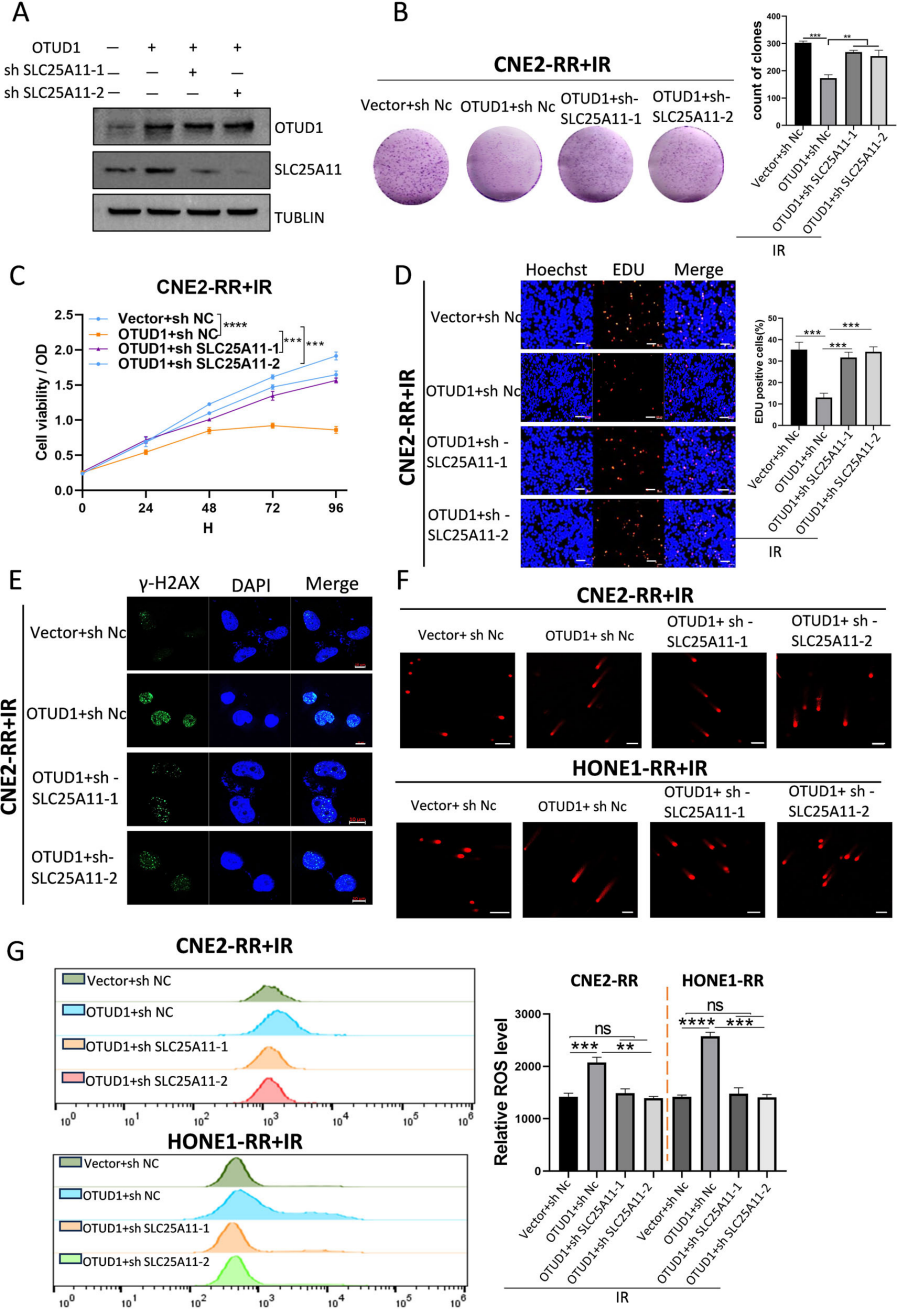
OTUD1 enhances radiosensitivity in radioresistant NPC cells through SLC25A11 stabilization. **A** WB analysis verifying OTUD1 overexpression and SLC25A11 knockout in NPC cell lines. **B**–**D** Cell viability and proliferation assays showing that the radiosensitizing effect of OTUD1 overexpression is reversed upon SLC25A11 knockout. Scale bars, 20 μm. **E**, **F** Immunofluorescence analysis for γ-H2AX and comet assay results show that DNA damage induced by OTUD1 overexpression is reversed after SLC25A11 depletion. Scale bars, 10 μm and 100 μm. **G** ROS levels in NPC cells following IR treatment were measured, showing reversal of OTUD1-induced changes after SLC25A11 knockout. Data are presented as means ± S.D., with *P* values determined using the two-tailed Student’s *t*-test. *n* = 3 independent experiments.

To examine the effect of OTUD1 in enhancing radiosensitivity in radioresistant NPC cells through SLC25A11 stabilization, we established OTUD1-overexpressing cell lines. These were divided into groups with or without SLC25A11 knockdown, validated by western blotting (Fig. 4A). We examined their response to IR. The radiosensitizing phenotype driven by OTUD1 overexpression was reversed upon SLC25A11 knockdown (Fig. 4B–D and Supplementary Fig. S5A–D). Similarly, increased DNA damage observed in OTUD1-overexpressing cells was reversed following SLC25A11 depletion (Fig. 4E, F, and Supplementary Fig. S5E). Further analysis revealed that GSH and ROS levels in the cells were also reversed in the absence of SLC25A11 (Fig. 4G and Supplementary Fig. S5F). Upon OTUD1 knockdown, GSH levels were found to increase under irradiation, correlating with decreased apoptosis following OTUD1 depletion (Supplementary Fig. S5G). These findings establish SLC25A11 as a critical downstream effector of OTUD1 in promoting radiosensitivity by stabilizing ROS production. Knockdown of SLC25A11 reversed the effects of OTUD1 overexpression, confirming the essential role of SLC25A11 in mediating the radiosensitizing function of OTUD1.

### TFAP2C-mediated hypermethylation upregulates OTUD1

**Fig. 5:**
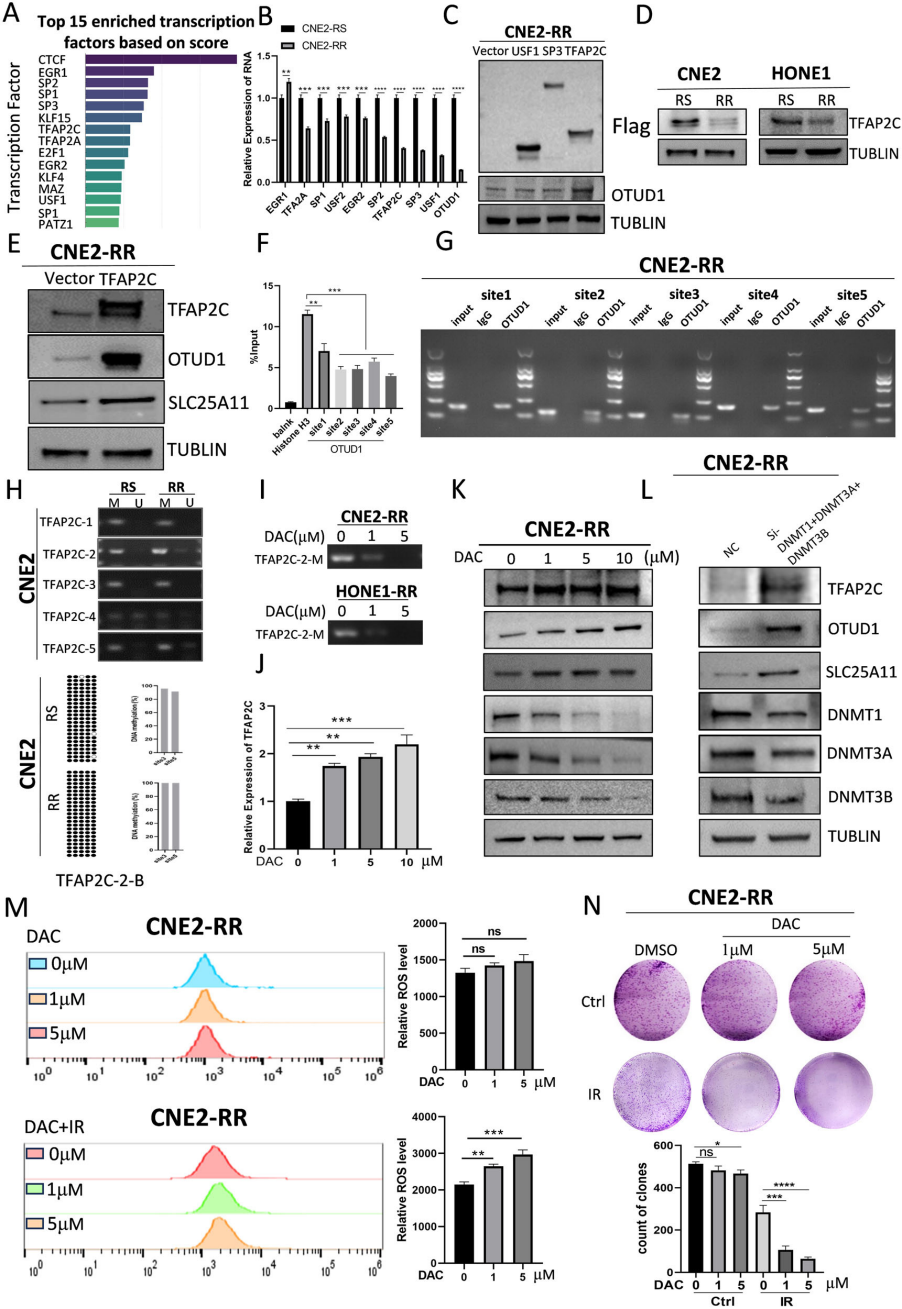
TFAP2C-Mediated Hypermethylation Upregulates OTUD1. **A**, **B** qPCR analysis of transcription factor expression across radiosensitive and radioresistant NPC cell lines, highlighting significant downregulation of TFAP2C, SP3, and USF1 in radioresistant cells. **C**, **D** Overexpression of TFAP2C leads to a significant increase in OTUD1 expression in both radiosensitive and radioresistant cells. **E** WB analysis shows the effect of TFAP2C overexpression and knockdown on OTUD1 and SLC25A11 protein levels. **F**, **G** ChIP assay results indicate the binding of TFAP2C to the OTUD1 promoter at several key sites, with different affinities. **H** MSP and BSP analyses show higher TFAP2C promoter methylation in radioresistant NPC cells, especially at site 2. In BSP, black circles indicate methylation, while white circles represent unmethylation. **I**–**K** Treatment with the methylation inhibitor DAC reduces TFAP2C methylation and increases TFAP2C and OTUD1 expression. **L** Knockdown of three DNA methyltransferases further validates the role of methylation in TFAP2C and OTUD1 regulation. **M** Detection of ROS shows enhanced ROS levels after DAC treatment under irradiation. **N** Clonogenic survival assays confirm that DAC and radiotherapy together increase radiosensitivity in radioresistant cells. Data are presented as means ± S.D. with *P* values determined using the two-tailed Student’s *t*-test; *n* = 3 independent experiments.

To elucidate the mechanisms underlying the downregulation of OTUD1 mRNA levels in radioresistant cells, we focused on its upstream transcription factors (Dataset). We conducted a comprehensive search using the AnimalTFDB4 database, ranking the top 10 potential transcription factors based on scores and enrichment levels, and validated them in radiosensitive and radioresistant cell lines (Fig. 5A). Among these, TFAP2C, SP3, and USF1 were markedly downregulated in radioresistant cell lines (Fig. 5B). Overexpressing TFAP2C had the most significant impact on OTUD1 levels (Fig. 5C), demonstrating a similar trend across cell lines (Fig. 5D). This suggests TFAP2C is a crucial upstream regulator of OTUD1. Further validation through TFAP2C overexpression and knockdown confirmed its regulatory effect on OTUD1 and SLC25A11 (Fig. 5E and Supplementary Fig. S6A). ChIP assays indicated that TFAP2C binds to the OTUD1 promoter at the top five ranked sites with varying affinities (Fig. 5F, G).

Next, we investigated the cause of TFAP2C and OTUD1 downregulation in radioresistant cell lines. Given the crucial role of methylation in radiation therapy [43, 44], we hypothesized that TFAP2C and OTUD1 expression could be influenced by methylation. Using MethPrimer, we identified methylation sites and designed primers to evaluate TFAP2C and OTUD1 methylation levels in radiosensitive and radioresistant cell lines (Supplementary Fig. S6B). While OTUD1 showed no methylation (Supplementary Fig. S6C), TFAP2C exhibited high methylation levels, particularly at site 2 in radioresistant cells (Fig. 5H and Supplementary Fig. S6D). Treatment with the methylation inhibitor DAC reduced TFAP2C methylation, leading to increased TFAP2C expression, which subsequently upregulated OTUD1 levels (Fig. 5I–K).

Knocking out three key DNA methyltransferases also increased TFAP2C and OTUD1 expression (Fig. 5L). Additionally, DAC treatment enhanced ROS production under irradiation, while clonogenic assays demonstrated that combining DAC with radiotherapy increased radiosensitivity in radioresistant cells (Fig. 5M, N). Thus, TFAP2C is an upstream regulator of OTUD1, with its expression modulated by DNA methylation. Enhancing TFAP2C expression can elevate OTUD1 levels, ultimately increasing radiosensitivity in NPC cells.

### OTUD1 enhances radiosensitivity of NPC cells in *vivo* by stabilizing SLC25A11

**Fig. 6:**
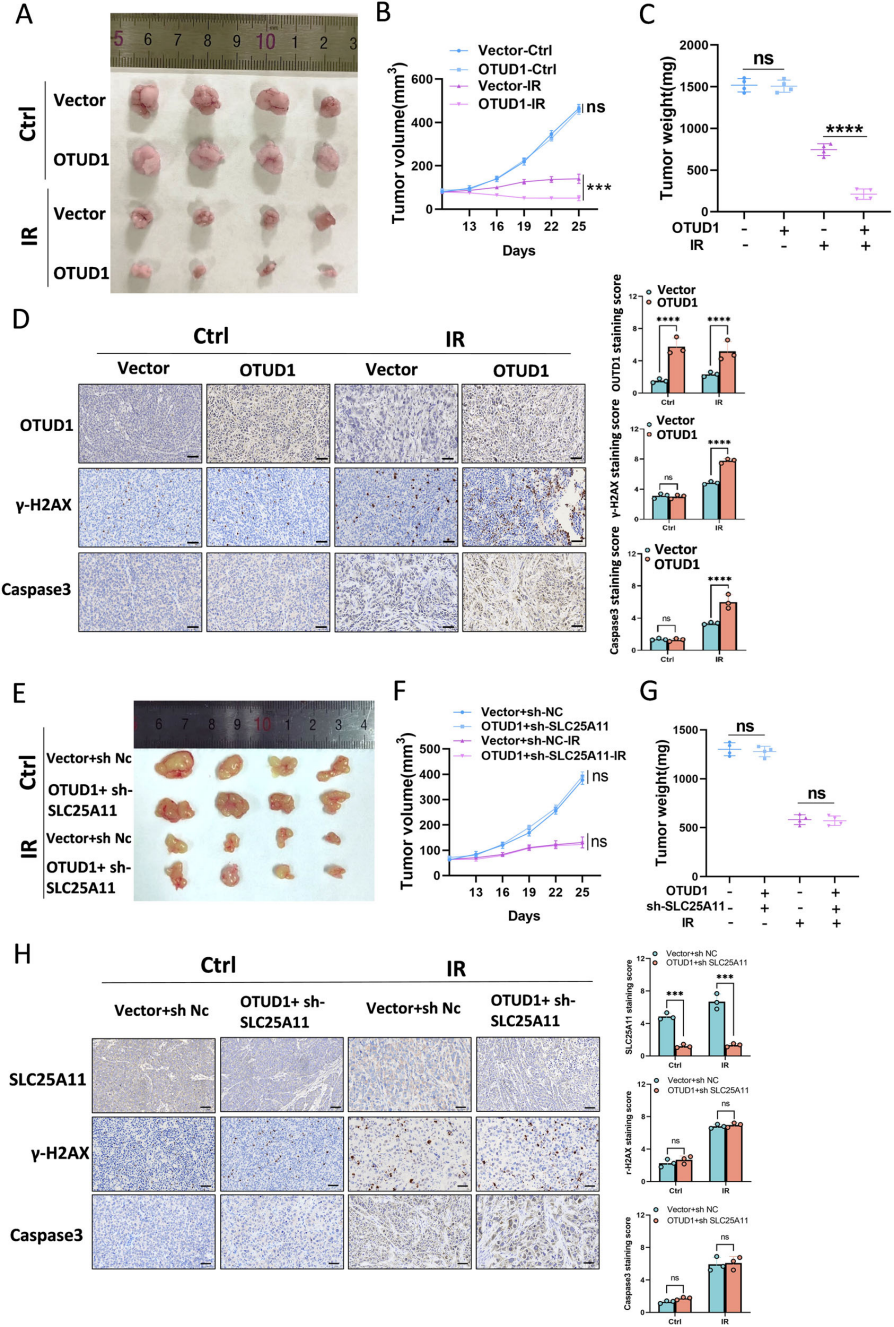
OTUD1 Enhances Radiosensitivity of NPC Cells In Vivo by Stabilizing SLC25A11. **A**–**C** Tumor growth curves and tumor weights in the OTUD1-overexpressing group vs. control, showing significantly slower growth in the OTUD1 group post-irradiation. **D** Immunohistochemistry staining indicating increased apoptosis and DNA damage in OTUD1-overexpressing tumors following RT. Scale bars, 100 μm. **E**–**G** Tumor growth and radiosensitivity in OTUD1-overexpressing and SLC25A11 knockdown models, demonstrating that silencing SLC25A11 impairs OTUD1’s radiosensitizing effect. **H** Immunohistochemical staining revealed that after radiotherapy (RT), knocking down SLC25A11 reversed the enhanced tumor apoptosis and increased DNA damage induced by OTUD1 overexpression. Scale bars, 100 μm. Data are presented as means ± S.D., with *P* values determined using the two-tailed Student’s *t*-test. *n* = 3 independent experiments.

To investigate whether OTUD1 affects radiosensitivity in vivo, we established xenograft mouse models using 2 × 10^6^ luciferase-expressing radioresistant NPC cells (CNE2RR-Vector and CNE2RR-OTUD1). These cells were injected subcutaneously into nude mice. After 10 days, mice were randomly divided into two groups: control and radiotherapy. The radiotherapy group received local irradiation at 12 Gy, delivered in two fractions (Supplementary Fig. S7A). Tumor diameters were measured every 3 days. Without irradiation, no significant differences were observed in tumor size, growth rate, apoptosis levels, or weight between the OTUD1 overexpression and control groups. However, post-irradiation, OTUD1-overexpressing tumors exhibited significantly slower growth than the control group (Fig. 6A–C). IHC and TUNEL assays demonstrated that OTUD1 overexpression significantly enhanced apoptosis and DNA damage in response to radiotherapy (Fig. 6D and Supplementary Fig. S7B). To further explore the role of the OTUD1-SLC25A11 axis in radiosensitivity, we created in vivo models with CNE2RR cells transfected with OTUD1, with or without SLC25A11 knockdown. In the absence of IR, upregulating OTUD1 or silencing SLC25A11 did not significantly affect tumor growth. However, during radiotherapy, silencing SLC25A11 impaired the radiosensitizing effect of OTUD1 (Fig. 6E–H).

These results suggest that OTUD1 enhances the radiosensitivity of NPC cells by stabilizing SLC25A11, with SLC25A11 knockdown reversing the effects of OTUD1 overexpression. This confirms the role of SLC25A11 as a key downstream effector in OTUD1-mediated radiosensitization.

### DAC combined with radiotherapy promotes radiosensitivity in NPC by enhancing OTUD1 expression

**Fig. 7:**
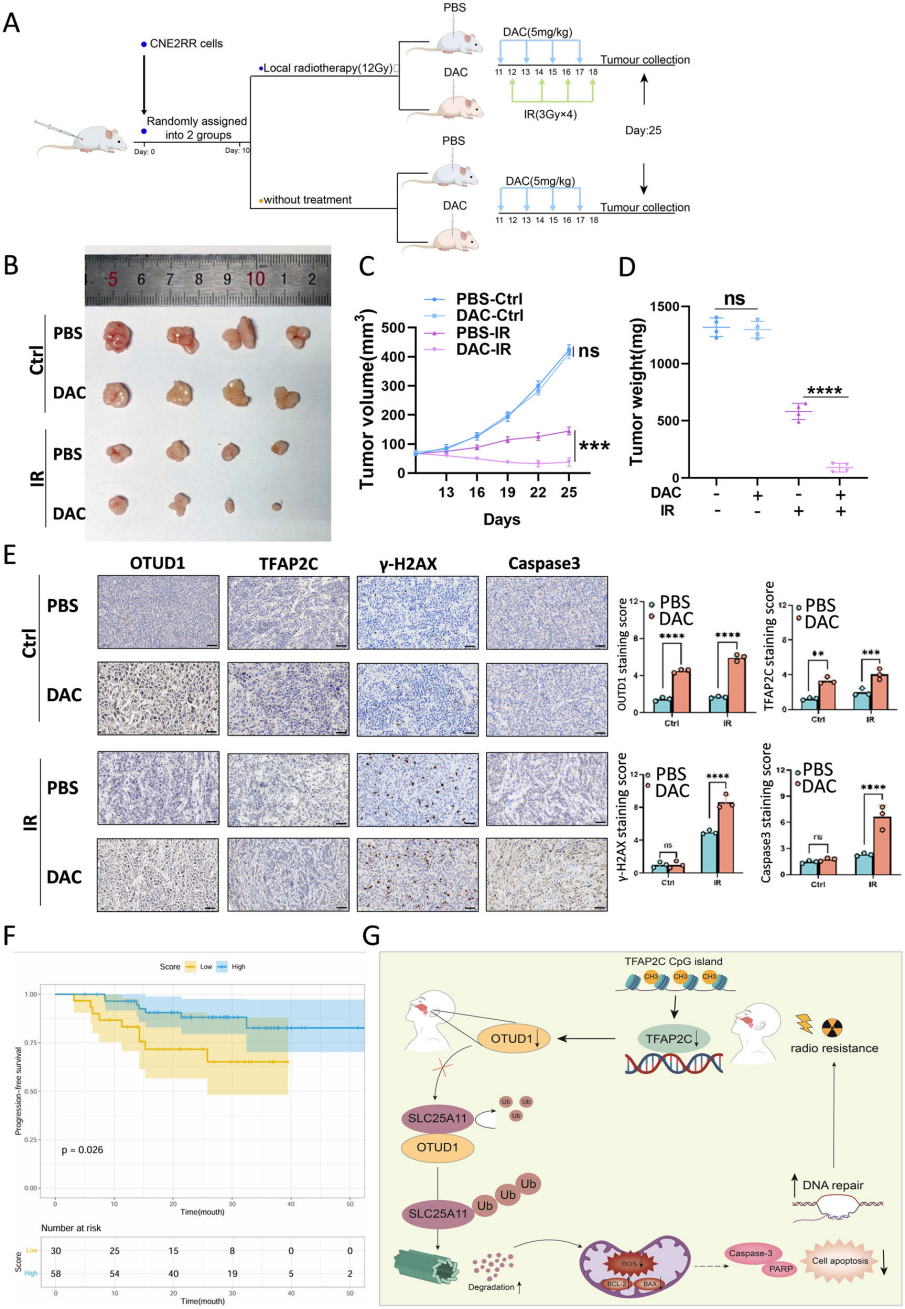
DAC combined with radiotherapy promotes radiosensitivity in NPC by enhancing OTUD1 expression. **A** Schematic of the experimental setup for in vivo xenograft mouse models. CNE2RR cells (2 × 10^6^) were injected subcutaneously into nude mice, which were divided into two groups: DAC treatment alone and DAC combined with radiotherapy (RT). **B**–**D** Tumor growth curves and weights were measured over time in both treatment groups. The combination of DAC and RT significantly inhibited tumor growth and increased apoptosis compared to DAC alone. **E** Immunohistochemical analysis of tumor tissue, demonstrating enhanced apoptosis and DNA damage in the DAC + RT treatment group compared to RT alone. Scale bars, 100 μm. **F** The Kaplan–Meier survival curve shows the progression-free survival (PFS) of NPC patients, divided into low- and high-score groups according to the enrichment scores of the TFAP2C-OTUD1-SLC25A11 gene set. Data are represented as mean ± S.D., with *P* values determined using the two-tailed Student’s *t*-test; *n* = 3 independent experiments. **G** Schematic of the TFAP2C-OTUD1-SLC25A11 regulatory axis modulating radiosensitivity in nasopharyngeal carcinoma.

To investigate whether DAC enhances NPC radiosensitivity in vivo, we established a xenograft mouse model. Mice were injected subcutaneously with 2 × 10^6^ radioresistant NPC cells (CNE2RR). The mice were randomly divided into two groups: one group received DAC treatment combined with local radiotherapy, while the other received DAC alone (Fig. 7A). In the absence of radiation, low-dose DAC showed minimal effects on tumor size, growth rate, apoptosis, or tumor weight. However, when combined with radiotherapy, DAC significantly improved radiosensitivity, resulting in greater reductions in tumor size, growth rate, and weight while increasing apoptosis (Fig. 7B–D and Supplementary Fig. S7C).

To evaluate potential toxicity, histological examination (HE staining) of the heart, liver, spleen, lung, and kidney in the DAC-only group showed no significant damage (Supplementary Fig. S7D), confirming the non-toxicity of the DAC concentration used. Further IHC analysis revealed that DAC enhanced TFAP2C and OTUD1 expression in tumors (Fig. 7E), promoting both apoptosis and DNA damage. Additionally, markers for cell proliferation (TUNEL), apoptosis(Caspase3), and DNA damage (γ-H2AX) indicated that tumors in the DAC + radiotherapy group exhibited significantly higher levels of apoptosis than those treated with radiotherapy alone (Fig. 7E and Supplementary Fig. S7C). Furthermore, Kaplan-Meier analysis of NPC patients from the GEO database showed a significant association between the TFAP2C-OTUD1-SLC25A11 gene set expression and progression-free survival (PFS) (Fig. 7F). These findings demonstrate that DAC, when combined with radiotherapy, enhances the radiosensitivity of NPC tumors in vivo.

## Discussion

Radiotherapy is a primary treatment for nasopharyngeal carcinoma, but widespread radioresistance often leads to poor patient outcomes [1, 26]. DUBs have emerged as potential radiotherapy targets in cancer, though the mechanisms remain unclear. Understanding how DUBs contribute to radioresistance could reveal therapeutic targets to improve treatment efficacy.

Our analysis of NPC cells showed that OTUD1 is significantly downregulated in radioresistant cells, a finding confirmed in patient samples. Studies indicate that OTUD1 plays a crucial role in various cancers by stabilizing p53, promoting apoptosis through p21 and Mdm2 upregulation, and modulating the TGF-β and Hippo pathways [15, 34–36]. These insights underscore OTUD1’s potential impact on radiotherapy efficacy in NPC.

Previous studies have highlighted the role of various DUBs in tumor progression, with OTUD1 recognized as a key tumor suppressor. However, its role in NPC and radiotherapy remains unexplored. Our research found that OTUD1 stabilizes the mitochondrial protein SLC25A11, elevating ROS levels and inducing oxidative stress, which leads to tumor cell apoptosis. This ROS-induced apoptosis mechanism significantly enhances the radiosensitivity of radioresistant NPC cells. These findings uncover a crucial role of OTUD1 in modulating ROS levels and enhancing radiotherapy efficacy, offering a novel target for improving radiotherapy outcomes in NPC.

To understand the regulation of OTUD1, we explored its upstream mechanisms. While OTUD1 is key for radiosensitivity, its transcription is significantly reduced in radioresistant cells. We identified TFAP2C as a crucial transcription factor for OTUD1 expression. Additional analysis showed that TFAP2C is downregulated in radioresistant NPC cells due to high DNA methylation, resulting in lower OTUD1 levels and a radioresistant phenotype. This finding is the first to reveal the mechanism by which TFAP2C regulates OTUD1 in radiotherapy resistance, providing new insights into the role of epigenetics in radiotherapy resistance.

Given that TFAP2C downregulation is linked to DNA hypermethylation, we explored the impact of methylation on this process. DNA methylation, a common epigenetic modification, is often associated with gene repression. We hypothesized that modifying the methylation state could restore TFAP2C and OTUD1 expression, thus enhancing tumor cell radiosensitivity. To test this, we conducted combined experiments using DAC with radiotherapy, which demonstrated that DAC effectively reduced TFAP2C methylation levels, restored its expression, increased OTUD1 levels, and significantly enhanced radiosensitivity in NPC cells.

These findings suggest that DAC, by reversing the epigenetic silencing of TFAP2C and OTUD1, offers a promising strategy to overcome radiotherapy resistance. This therapeutic approach, involving deubiquitination pathways and epigenetic modification, aligns with emerging methods to boost radiotherapy efficacy through adjunctive treatments. Optimizing the dose, timing, and potential side effects of combining DAC with radiotherapy is crucial to maximize radiosensitization while minimizing toxicity. Although we demonstrated the efficacy of DAC combined with radiotherapy, its potential off-target effects on non-tumor tissues have yet to be fully evaluated. While no toxicity was observed in our model, further research is needed to confirm the safety of prolonged DAC use alongside radiotherapy.

In summary, Fig. 7G presents our model. We found that OTUD1 stabilizes SLC25A11, increasing ROS levels and inducing apoptosis to enhance radiosensitivity. Additionally, TFAP2C, an upstream transcription factor of OTUD1, is downregulated in radioresistant cells due to hypermethylation, resulting in reduced OTUD1 expression and radiotherapy resistance. Combining the methylation inhibitor DAC with radiotherapy restores TFAP2C and OTUD1 expression, significantly boosting radiosensitivity. This approach could improve radiotherapy outcomes for radioresistant NPC patients, offering a promising epigenetic-based therapeutic strategy.

## Conclusion

OTUD1 enhances NPC radiosensitivity by promoting ROS production and apoptosis, and TFAP2C regulates OTUD1 to stabilize SLC25A11. Moreover, combining DAC with radiotherapy significantly improves therapeutic outcomes. These findings provide insights into NPC radioresistance and suggest that using DAC in combination with radiotherapy to target the TFAP2C-OTUD1-SLC25A11 axis could be a promising strategy to overcome radioresistance.

## Supplementary information

Supplementary Material

Dataset 1

## Data Availability

The RNA-seq datasets and transcription factor analysis data related to this study can be accessed publicly via the AnimalTFDB4 database (AnimalTFDB4, https://guolab.wchscu.cn/AnimalTFDB4/#/). For other sequencing data, please refer to the supplementary data or contact the corresponding author at any time. The mRNA expression profiles of 113 nasopharyngeal carcinoma (NPC) patients used in this study are available in the Gene Expression Omnibus (GEO; https://www.ncbi.nlm.nih.gov/geo/) under accession code GSE102349. Among these patients, 88 have complete progression-free survival (PFS) data, making this dataset the only publicly available resource to date that includes prognostic information for NPC patients. The datasets used for survival analysis and gene set enrichment analysis can be accessed through the GEO database.
