# Supplementary material for: Combining decitabine with radiotherapy to enhance nasopharyngeal carcinoma radiosensitivity via the TFAP2C-OTUD1-SLC25A11 axis
Source: Cell Death Dis. 2025 Jul 15;16(1):525. doi: 10.1038/s41419-025-07858-9 (PMC12264102; doi:10.1038/s41419-025-07858-9)
Supplement: Supplementary file 1 — Supplementary Material [file 41419_2025_7858_MOESM1_ESM.pdf]

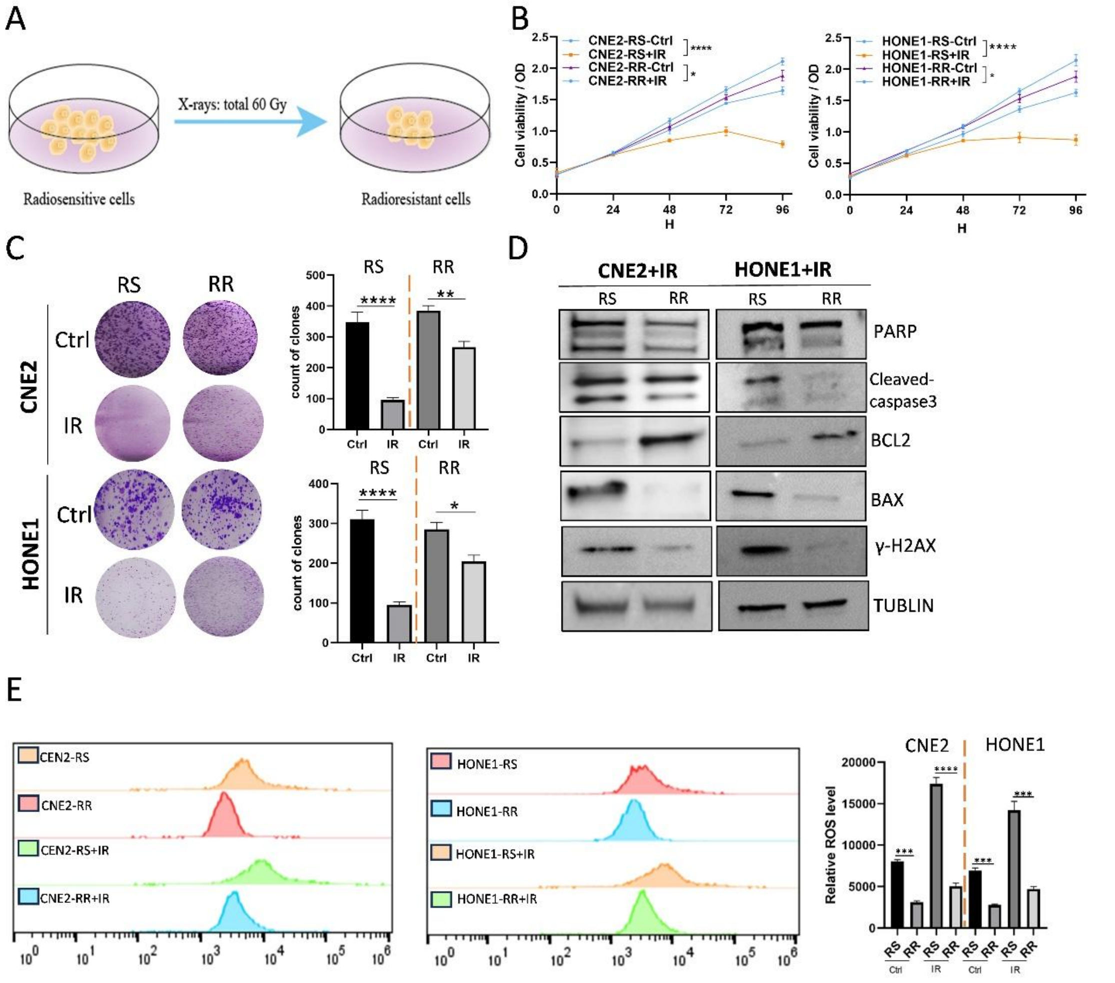

S1: Validation of Radiation-Resistant Cell Lines

(A) CNE2-RR and HONE1-RR radioresistant cell lines were generated from parental CNE2 and HONE1 cell lines through repeated low-dose irradiation. (B-D) Clonogenic survival, CCK8, apoptosis, and other assays confirmed that the radioresistance of CNE2-RR and HONE1-RR cell lines is higher compared to their parental cell lines. (E) ROS detection results showed that the ROS levels in radioresistant strains were lower than those in radiosensitive strains, consistent under both irradiation and non-irradiation conditions. Data are presented as means ± S.D., with P values determined using the two-tailed Student's t-test. n = 3 independent experiments.

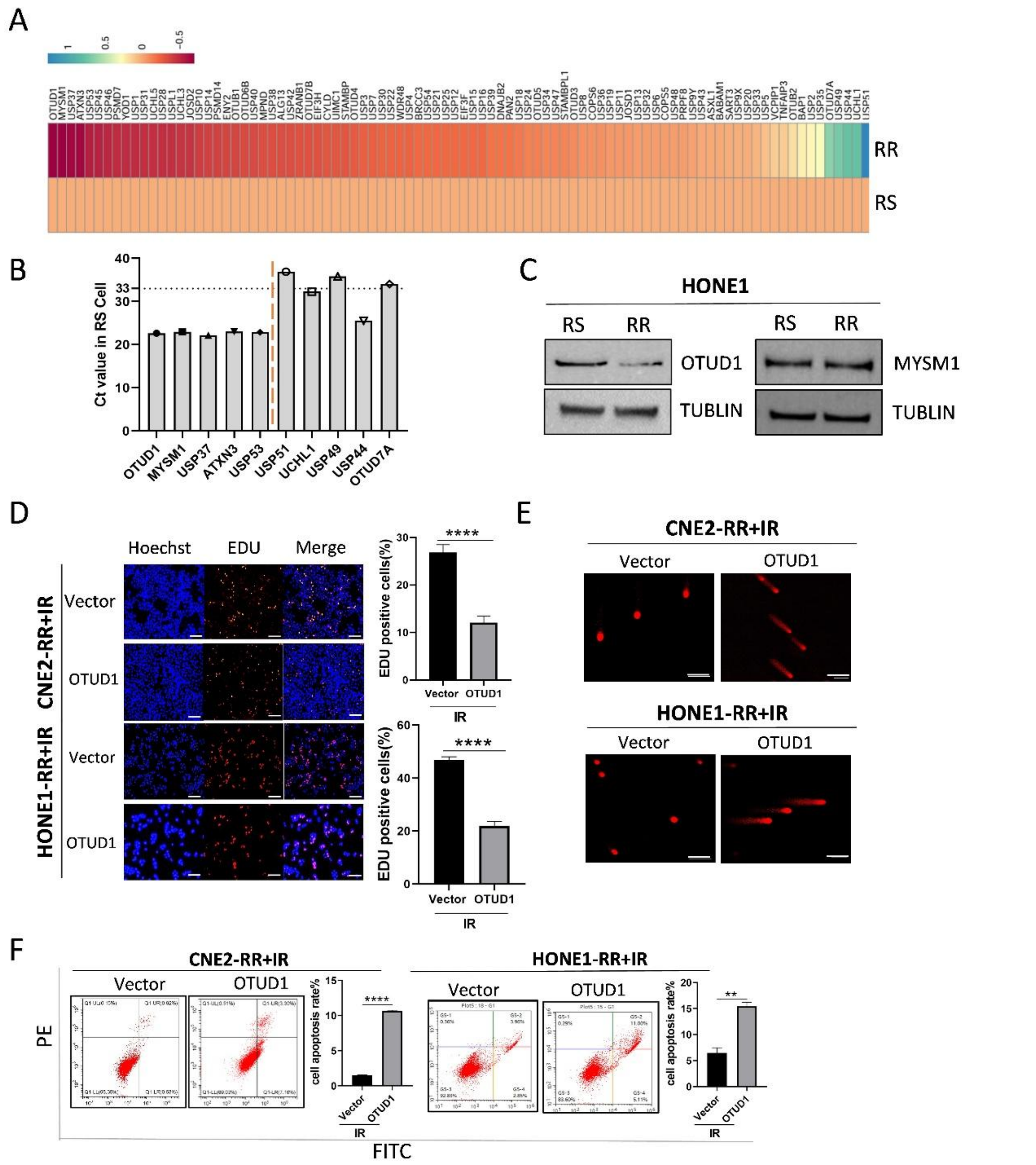

S2: OTUD1 promotes ROS accumulation and radiosensitivity in NPC cells

(A) Additional qPCR results of differentially expressed deubiquitinases. (B) CT values of the top five upregulated and downregulated deubiquitinases in radioresistant and radiosensitive NPC cells. (C) WB analyses of OTUD1 and MYSM1 expression in NPC cell lines. (D) EdU assay confirming impaired proliferation in OTUD1-overexpressing NPC cells post-irradiation. (E) Comet assay showing increased DNA damage in OTUD1-overexpressing cells. (F) Flow

cytometry analysis indicating elevated apoptosis in OTUD1-overexpressing cells after irradiation. Data are presented as means ± S.D., with P values determined using the two-tailed Student's t-test. n = 3 independent experiments.

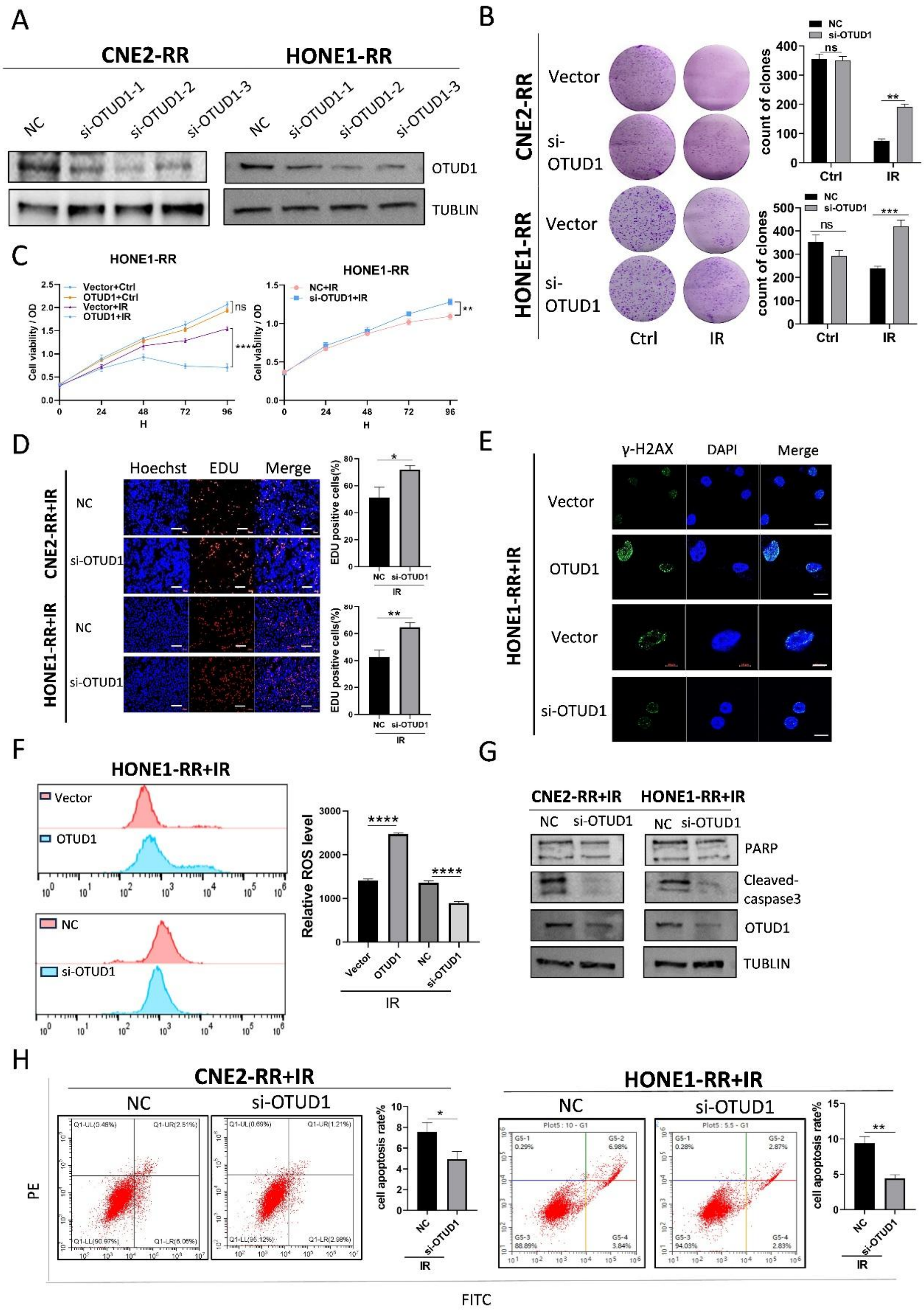

S3: OTUD1 Knockdown Reduces ROS Levels and Decreases Radiosensitivity

(A) Knockdown efficiency of OTUD1 validated by WB in radioresistant NPC cell lines. The sequence with the best knockdown effect was selected for further study. (B-D) The effect of OTUD1 knockdown on cell survival and proliferation under radiotherapy conditions. Knockdown of OTUD1 improved cell survival and proliferation compared to control. (E) Immunofluorescence staining of γ-H2AX was used to assess DNA damage effects following OTUD1 knockdown under radiotherapy conditions. (F) Measurement of ROS levels in radioresistant NPC cells after OTUD1 knockdown under irradiation conditions. (G) WB analysis confirming OTUD1 knockdown and its impact on apoptosis-related protein expression under irradiation conditions. (H) Flow cytometry analysis of the apoptosis rates in OTUD1 knockdown radioresistant NPC cells under irradiation conditions. Data are presented as means ± S.D., with P values determined using the two-tailed Student's t-test. n = 3 independent experiments.

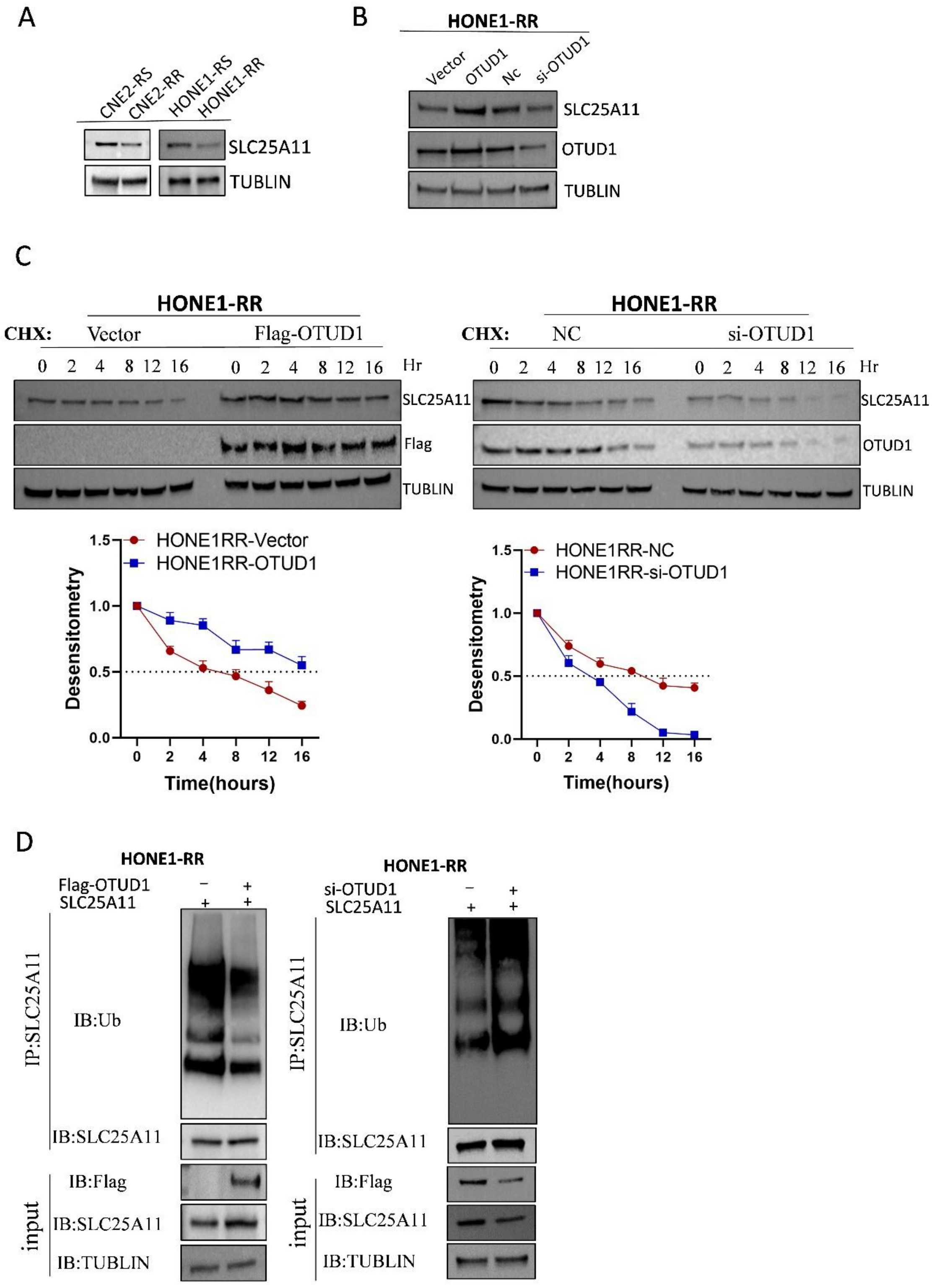

S4: OTUD1 stabilizes SLC25A11 by reducing its ubiquitination

(A) Western blot analysis was performed to assess the expression levels of SLC25A11 in radioresistant NPC cells. (B) WB analysis showing that overexpression of OTUD1 enhances

SLC25A11 expression, while silencing OTUD1 inhibits SLC25A11 expression in HONE1-RR cells (C) Overexpression of OTUD1 extended the half-life of endogenous SLC25A11, while silencing OTUD1 shortened its degradation half-life in HONE1-RR cell. (D) Ubiquitination assay to assess the polyubiquitination of SLC25A11 in HONE1-RR cell, with OTUD1 overexpression or silencing, using immunoprecipitation and WB analysis. Data are presented as means ± S.D., with P values determined using the two-tailed Student's t-test. n = 3 independent experiments.

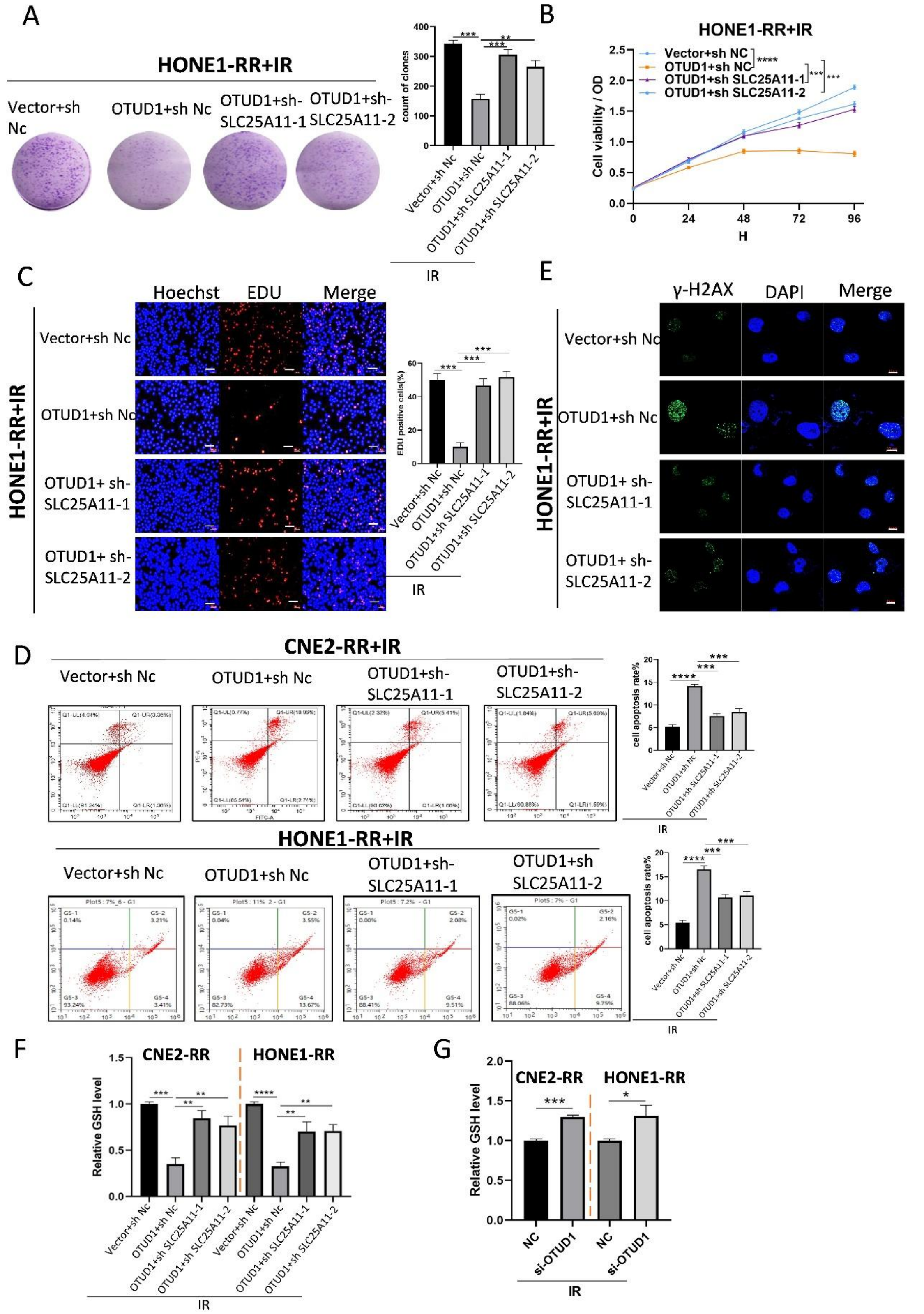

S5: OTUD1 Enhances Radiosensitivity in Radioresistant NPC Cells through SLC25A11 Stabilization

(A-C) Clonogenic assays, CCK8, and EdU assays showing the reversal of OTUD1-enhanced radiosensitivity upon SLC25A11 knockout. (D) Flow cytometric analysis of apoptosis levels

showing that OTUD1-induced apoptosis is significantly reduced following SLC25A11 knockout. (E) Immunofluorescence analysis for γ-H2AX show that DNA damage induced by OTUD1 overexpression is reversed after SLC25A11 depletion. (E) GSH levels in NPC cells following IR treatment were measured, showing reversal of OTUD1-induced changes after SLC25A11 knockout. (F) OTUD1 knockdown under irradiation increases GSH levels, which aligns with a reduction in apoptosis in nasopharyngeal carcinoma cells. Data are presented as means ± S.D., with P values determined using the two-tailed Student's t-test. n = 3 independent experiments.

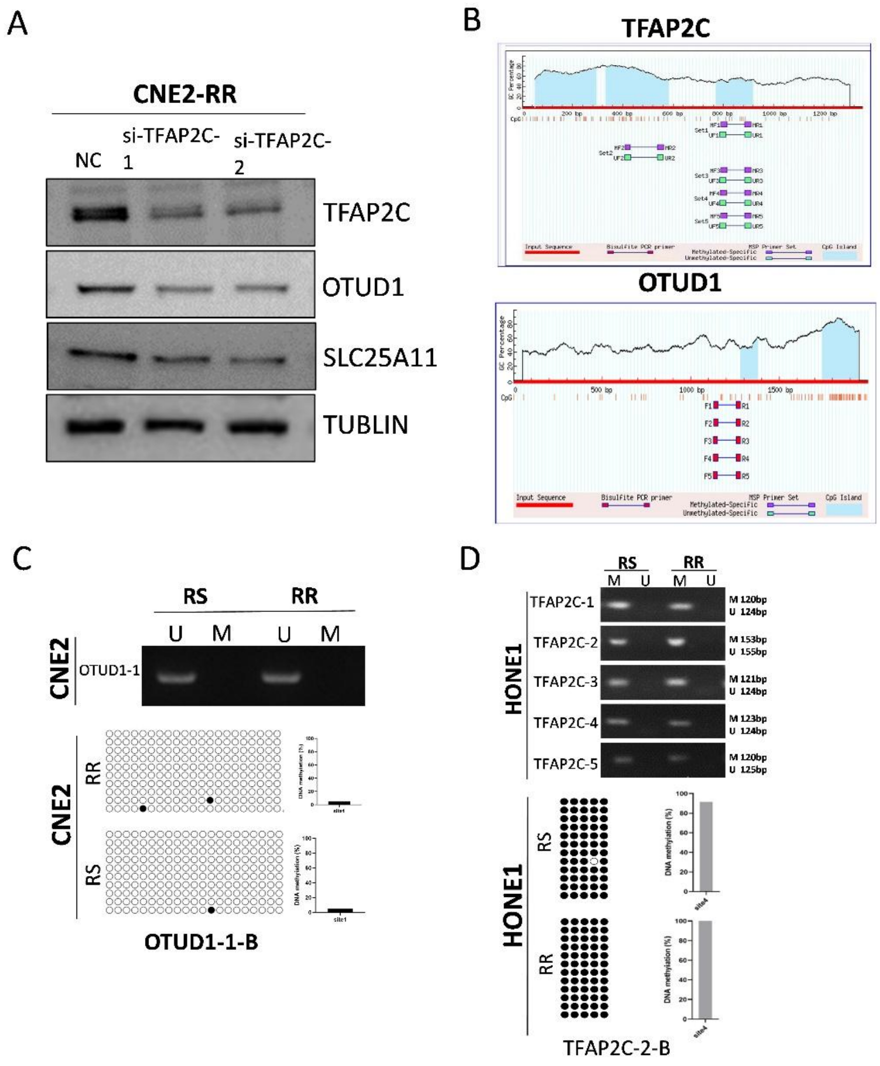

S6: TFAP2C-Mediated Hypermethylation Upregulates *OTUD1*

(A) Western blot showing the effect of TFAP2C knockdown on OTUD1 and SLC25A11 expression. (B) MethPrimer analysis reveals the identified methylation sites in the TFAP2C and OTUD1 promoter region. (C) MSP results indicate no significant methylation at the OTUD1 promoter region in NPC cells. (D) BSP analysis confirms higher methylation levels at site 2 of TFAP2C in radioresistant cells compared to radiosensitive cells. Data are presented as means ± S.D., with P values determined using the two-tailed Student's t-test. n = 3 independent experiments.

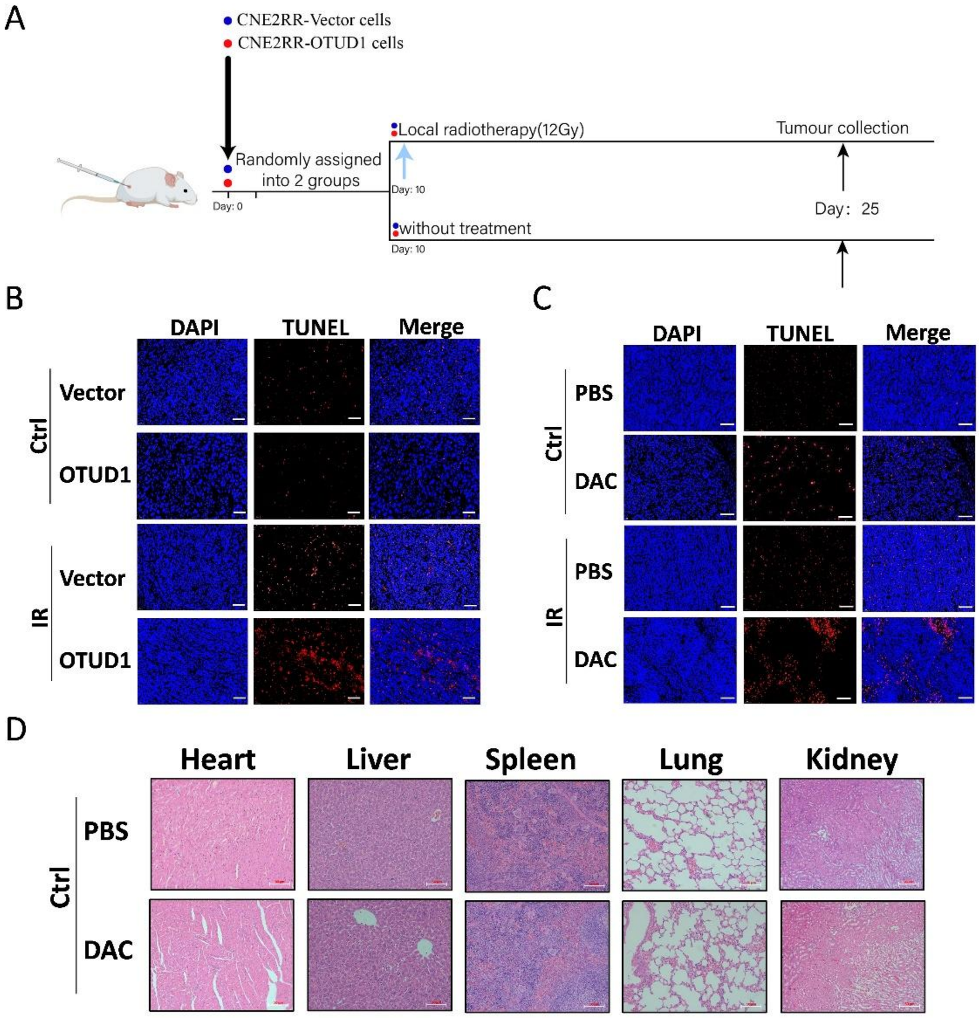

S7: OTUD1 Enhances Radiosensitivity of NPC Cells In Vivo by Stabilizing SLC25A11

(A) Experimental design for in vivo xenograft model using luciferase-expressing radioresistant CNE2RR-Vector and CNE2RR-OTUD1 cells. After tumor establishment, mice were divided into control and radiotherapy (RT) groups, with tumors in the RT group receiving a total of 12 Gy in two fractions. (B) TUNEL assay demonstrating that OTUD1 overexpression combined with RT enhances tumor apoptosis. (C) TUNEL assay showing that DAC combined with RT increases tumor apoptosis compared to DAC alone. (D) H&E staining of major organs (heart, liver, spleen, lung, and kidney) from the DAC-alone group, showing no significant toxicity. Data are presented as means ± S.D., with P values determined using the two-tailed Student's

t-test. n = 3 independent experiments.

Table

Table 1. Primer sequences used for quantitative real-time PCR analysis in this study	
DNMT1-F	AGGTGGAGAGTTATGACGAGGC
DNMT1-R	GGTAGAATGCCTGATGGTCTGC
DNMT2-F	CACAAGTGGTGGCTGCCATTGA
DNMT2-R	GTCAAACTCTTCGAGTGTAATGCC
DNMT3A-F	CCTCTTCGTTGGAGGAATGTGC
DNMT3A-R	GTTTCCGCACATGAGCACCTCA
DNMT3B-F	TAACAACGGCAAAGACCGAGGG
DNMT3B-R	TCCTGCCACAAGACAAACAGCC
UCHL1-F	CAGTTCAGAGGACACCCTGCTG
UCHL1-R	CCACAGAGCATTAGGCTGCCTT
OTUB2-F	GCTGGCTTTGAGGAGCACAAGT
OTUB2-R	CTGGTCGTTGAACACCTTCAGC
PSMD14-F	GTCAGTGTGGAGGCAGTTGATC
PSMD14-R	CCACACCAGAAAGCCAACAACC
USP18-F	TGGACAGACCTGCTGCCTTAAC
USP18-R	CTGTCCTGCATCTTCTCCAGCA
STAMBPL1-F	GGCTTTCATCCACACACCAAGG
STAMBPL1-R	GAGTAGGTGTCTGATGTTGACGG
USP28-F	GACCTGTCTTCAGAGATGGAGG
USP28-R	TGACGAAGGAGAGGATCGCAGT
USP21-F	GTTCTATGGCAGATTCCTGGCTG
USP21-R	GCAGCTTCTAGGCACTCATGCA
BRCC3-F	CGGGTACTCTACACTTGCTTCC
BRCC3-R	CCTCAATGGAGATGTGCTGGCT
UCHL3-F	CAAACAATCAGCAATGCCTGTGG
UCHL3-R	GGCTCATTGACACAGATTCCTCC
UCHL5-F	TGTGGTTCAGGACTCCCGACTT
UCHL5-R	CGCCTAAATGGACATCCTGGTG
USP35-F	AGAGAACTTCCTCTCCGCATCC
USP35-R	CTGGACTGCTTGAGTTTCTGGC
COPS6-F	AGCGCCATCAAGATGCTGCACA
COPS6-R	GACAGTGACACAGAGCATAGGC
USP39-F	GACGAGAAGGAGCAGCTCATCA
USP39-R	TGGAGGCAACTTGGTAAGCTGG
YOD1-F	CCATTCTGGAAGACTTGCCCATC
YOD1-R	ACCACGGTTCTGGTAAGCACAG
PSMD7-F	GATGTGAAGCCGAAGGACCTAG

PSMD7-R	TCCTCAGCTTCCTCTGCTCCAA
USP14-F	GGGAAATGGCTTCAGCGCAGTA
USP14-R	CACCTTTCTCGGCAAACTGTGG
STAMBP-F	CCTCATCACACTGGGCTGGATT
STAMBP-R	GGCTACTGACTCTGGCAACATC
COPS5-F	CCAGGAACCATTTGTAGCAGTGG
COPS5-R	GTCTGGTACTCAGAAGGTCCTTC
USP49-F	GGAGAATCTACGCTTGTGACCAG
USP49-R	CGGAGAACCTGAGGTAGTCTGT
USP43-F	CAGTTCTACACCAAGGAGGAGC
USP43-R	GATGTCAGGCAGCGTCCACAAA
EIF3H-F	GATTCAGCCGTGAAGCAAGTGC
EIF3H-R	CCAGACCCAAAAGCACTCCTTG
USP37-F	TCTCTATTGACCTTCCTCGTAGG
USP37-R	TGCCTGACAAGAGCACACTTCC
USP5-F	GAAGTGTTCCGCTTCTTGGTGG
USP5-R	TTGCCGCTTCTTCTCCTCGTAC
OTUD6B-F	CTGCTGAGAAGGCATCGCAAAG
OTUD6B-R	GCCACATCTTCGGTGAGTTGCT
USP10-F	AAATGCCACCGAACCTATCGGC
USP10-R	CAGCCATTCAGACCGATCTGGA
USP48-F	GTCCTCTACATCGTGTCTCAGTT
USP48-R	GGGACACAAAAGAGCACTGTTCC
OTUB1-F	GCTGGATGACAGCAAGGAGTTG
OTUB1-R	CTTCTCCACCTGCTCAATCAGG
USP42-F	ACAGCGACAGTGACCCGAAAGA
USP42-R	GACAGCAAAGGAGCAGGCATCA
MYSM1-F	CGCCAAGCTAAAACACCAGAACG
MYSM1-R	AACATCTCCACAGTTCTTCAGGC
USP45-F	CTTCACAGCATGAAGGAGACTGA
USP45-R	CTTCTGTCCTCACTGCATCCAG
PAN2-F	TGTTGGACCTCTCTCGTGGTGA
PAN2-R	CCTCATCTGAGTCAGCCAGGAT
ALG13-F	GTAGAGGAACGGTGGTACCTGA
ALG13-R	CAGAGTCTCCAAACAGCTTCCTG
USP31-F	CCTGTATGCTGTGTGCAATCACC
USP31-R	TCGCTGTCATCGAAGCAGTACC
USP1-F	GCTCTAAAGGATGAAGCCAATCAA
USP1-R	ACTAGCCTGGAGCTGTTCAACC
USP40-F	CAGAAAGCGTGTGGGATTTGACC
USP40-R	GTGAAGTCCTGCTGGTACAAGC
BABAM1-F	CCACAATGGCACTGAGGAGAAG
BABAM1-R	GCCACCTCATACTTGTAGCTGG

USP36-F	AGCAGATGTCCTGAGTGGAGAG
USP36-R	GATGTTCTGTGGATGGTGAAGCG
SART3-F	GGAGATTTGGCAGGCATACCTTG
SART3-R	CTCTTCCACCTCCTGCTTCAGA
VCPIP1-F	TGGTCTTATGCAAAGGGACTTCC
VCPIP1-R	GGTTTTGCCAGGCAGATGTGGA
ENY2-F	GCTGGAAGGATCAGTTGAAGGC
ENY2-R	GCTCCTTCTTTACACTGTCAGGT
USP9X-F	GTGTCAGTTCGTCTTGCTCAGC
USP9X-R	GCTGTAACGACCCACATCCTGA
USP32-F	CATGCCATCTCTTCCTGACAGC
USP32-R	CAATGGCATTCCAAAGAGGCTGG
USP24-F	TTCTGCTCGGATCACCAAGTGC
USP24-R	CTGCACATCTGGATGCGCTGAT
USP7-F	GTCACGATGACGACCTGTCTGT
USP7-R	GTAATCGCTCCACCAACTGCTG
USP20-F	CTTTGACGGCTCCATTCTCAGC
USP20-R	CTCCTTTCCAGGAATGGGCAGT
USP3-F	ATGCGCTACCTTTTGGACCACC
USP3-R	CCGTGACAACAGTAGATGCTCC
OTUD5-F	CAGGCTACAACAGTGAGGACGA
OTUD5-R	GAAGCCCTTCTTGTCTCGTAGG
DNAJB2-F	GGAGATTTACGACCGCTATGGC
DNAJB2-R	GCAAAAGGGTCTCCACTCCCAA
USP34-F	CGACTTAGATGCCTTGGCAAGAC
USP34-R	GGAGTCCTGTAAGCCCATCATC
USP46-F	GGCTCTTCAACACCTCCAGTGA
USP46-R	GCCAGAAGCCGTGACTTTTCAC
ASXL1-F	CGGCTTGAAGATCGTCAGTCCT
ASXL1-R	GGCTGACCTTTAACCACCCAGG
OTUD3-F	AGCACTACGACAGTGTTCGGAG
OTUD3-R	GGTCGTCTTCAGAGTCCATTCC
USPL1-F	AATGTCATCCCTGAGTGGCACC
USPL1-R	GCAAGTCATTCTGTGGTAAGCCT
JOSD2-F	GTGGACGGTGTCTACTACAACC
JOSD2-R	CACTACCAGCAGCACCTCGCA
USP9Y-F	GAGGTGGAAAGTTGTTTGCCTGG
USP9Y-R	TGCTCCATCTCTAAGAGGTGGC
USP15-F	CCCTACACTGCTGTAAGGACCA
USP15-R	GAGGGAAGTTCTTGATCCTGGC
BAP1-F	GAGGATGACGTGCAGAACACCA
BAP1-R	CTCAGCCAAGACGTTGATGGTG
JOSD1-F	GTCTTCCAGGACAGCAATGCCT

JOSD1-R	CCTTTGGTCTGAAGTGCTGCCA
USP22-F	CTACCAGGAGTCCACAAAGCAG
USP22-R	CACATACGTGGTGATCTTCCGC
EIF3F-F	CTCACTGTGGACACAAGTCTCC
EIF3F-R	TCCGATGCGTTCAGTGTCGTAG
USP19-F	GCTGCTATCCTCAGAGTTGGCT
USP19-R	TCATCCTCCGACTGTTGCTTCC
UIMC1-F	GCTCTTGAGGAAAGCCATTGCTG
UIMC1-R	TTCAGTGAGCCCAGAGTCTGTG
USP38-F	TGTGAGACCTGGTGCTCTTGCA
USP38-R	GGCTGTACTTGAAGGCAGACCA
USP4-F	GCAGCCACTATTGCTTTCTGTCC
USP4-R	CTGCCAAACTCATCAGGTAAAGG
USP33-F	GTCAAAGCAGGATCATGTGGCG
USP33-R	CCAAAACCAGCTAGGGACACATG
OTUD7B-F	TCTCAGAGGCTGCTTCCTTTGG
OTUD7B-R	CGCCTTTTCAACGCTTCCTTCTC
MPND-F	ACGCACAGATGGACTACCAGCT
MPND-R	AGGAGGCATCACCCAGAAAGGT
USP16-F	CTATGCCAAGGCAAGAACCGCA
USP16-R	GTCGCTGATGTGAAACCACTGC
PRPF8-F	TAAGTGCCGCACCAGCTATGAG
PRPF8-R	ACTCATCGTCCACACGCAGGAA
USP47-F	CTGCTGTTGGAGACGAGAAAGC
USP47-R	GCACGAACAGTTATAGGAGCAGC
USP30-F	GGTCTGCAAACACTGTGAACACC
USP30-R	AAGGCAGTGGTCCAGGGTCAAT
WDR48-F	GTGAATCCAATGGATGAAGAGGAA
WDR48-R	AACAGTGTGCGACCTCCAGCTT
USP11-F	GCTGATGTCTTCAGTCACCGCT
USP11-R	GGAACCACGATGTCCTCTCTTG
USP8-F	GATCGTACCAGGACTGCCTTCA
USP8-R	GCAGATGAAGGAGCCATTTCCG
OTUD4-F	CTAACTCCTGCGGTGCCTTCTT
OTUD4-R	GCTGAATCAGGTCCAGTGGTCA
ATXN3-F	TCGGAAGAGACGAGAAGCCTAC
ATXN3-R	AAGTGCTCCTGAACTGGTGGCT
USP13-F	CCTCTAACAGGCAGCAAGATGC
USP13-R	GCACTGAATGCGTTCTTCCACC
ZRANB1-F	CGCTTGCTGAATCGTCCTTCTG
ZRANB1-R	GTTCAGGACACACCATTGCTGG
USP6-F	CGGGCAAAGTTAGACCTTCCAG
USP6-R	TCCGTTGCTCAAGTGATGGACG

CYLD-F	GGTAATCCGTTGGATCGGTCAG
CYLD-R	AGTGCCTCTGAAGGTTCCATCC
USP53-F	CAGCCAACATTATTGTGCCTTTGC
USP53-R	GGCTGAAAGTGGCATCGAATGC
USP25-F	GTTCTATGGCAGATTCCTGGCTG
USP25-R	GCAGCTTCTAGGCACTCATGCA
USP51-F	GTTGCCAAAGCTACCAGGAGTC
USP51-R	GCCTACATGCTCAAACCGCTTG
USP54-F	GAGTTAGAGGCAGCGAAAGGGT
USP54-R	TCTTGCAGGGACCTCTCAAAGC
USP12-F	AGACCTTTCTGTTGACGTGGAAC
USP12-R	GTGTGCTTCCTGTTTGCTGCGA
OTUD7A-F	CCTGACGGATTCTGAGCACAAG
OTUD7A-R	GTTCAGCTTGGCTTCTAGCGAC
USP44-F	CTACCTCAGGTTCTCAGACTGC
USP44-R	TCTGAGGGATTTCAGGGTCTCC
USP2-F	GAGATACGCACCGCGCTTTGTT
USP2-R	GGTTGGACTTAGGTCTCAGTGTC
TNFAIP3-F	CTCAACTGGTGTCGAGAAGTCC
TNFAIP3-R	TTCCTTGAGCGTGCTGAACAGC
OTUD1-F	CAGTTGGCTCAGTAACGGACAC
OTUD1-R	GATTTGGCAAGTTCTTCGTCGCG
SLC25A11-F	ATCAGCGGTCTTGTCACCACTG
SLC25A11-R	GCGGACAACTTTGAACAGCACG
SP1-F	ACGCTTCACACGTTCGGATGAG
SP1-R	TGACAGGTGGTCACTCCTCATG
SP2-F	GGAAGAGGTTCACACGGAGTGA
SP2-R	CAAGTTCTTCGTGACCAGGTGG
SP3-F	TGTCCCAACTGTAAAGAAGGTGG
SP3-R	CTCCAGAATGCCAACGCAGATG
USF1-F	GCTCTATGGAGAGCACCAAGTC
USF1-R	AGACAAGCGGTGGTTACTCTGC
USF2-F	GGATGTGCTTCAGACAGGAACAC
USF2-R	CTTCGTTGTGCTGGGCTCTTCT
TFAP2C-F	CACCTGTTGCTGCACGATCAGA
TFAP2C-R	AGGAGCGACAATCTTCCAGGGA
TFAP2A-F	GACCTCTCGATCCACTCCTTAC
TFAP2A-R	GAGACGGCATTGCTGTTGGACT
EGR1-F	AGCAGCACCTTCAACCCTCAGG
EGR1-R	GAGTGGTTTGGCTGGGGTAACT
EGR2-F	CCTTTGACCAGATGAACGGAGTG
EGR2-R	GAAGGTCTGGTTTCTAGGTGCAG

Table1： The table lists the forward and reverse primer sequences designed for target gene

quantification by real-time PCR. All primers were synthesized by shenggong Biotech (Shanghai, China). Gene-specific amplification was validated by melting curve analysis.

**Table 2. Antibodies and chemical reagents used in this study**		
**Reagent Source**	**Catalog Number**	**Supplier**
Antibodies		
tublin	AC015	ABclonal, China
Cleaved PARP	CST5625	Cell Signaling Technology, USA
Cleaved caspase 3	CST9661	Cell Signaling Technology, USA
MYSM1	DF7188	Affinity Biosciences,China
OTUD1	NBP2-85429-0.1ml	Novus, USA
P62	18420-1-AP	Proteintech, China
GPX4	67763-1-Ig	Proteintech, China
BCL2	CST15071	Cell Signaling Technology, USA
P-MLKL	AP1173	ABclonal, China
LC3	2775S	Cell Signaling Technology, USA
BAX	CST2772	Cell Signaling Technology, USA
γ-H2AX	ab81299	abcam, Britain
Flag	AE005	ABclonal, China
SLC25A11	A8163	ABclonal, China
HA	AE008	ABclonal, China
Ub	10201-2-AP	Proteintech, China
TFAP2C	A7739	ABclonal, China
DNMT1	24206-1-AP	Proteintech, China
DNMT3A	20954-1-AP	Proteintech, China
DNMT3B	A11079	ABclonal, China
HRP Goat Anti-Rabbit IgG (H+L)	AS014	ABclonal, China
HRP Goat Anti-Mouse IgG (H+L)	AS003	ABclonal, China
Anti-mouse IgG (H+L), F(ab')2 Fragment (Alexa Fluor® 647 Conjugate)	CST4410	Cell Signaling Technology, USA
Drug		
DAC	GC15255	GLPBIO, China
CHX	S7418	Selleck, USA
MG132	S2619	Selleck, USA

VAD	GC12861	GLPBIO, China
Nec1	S8037	Selleck, USA
Necr	S8251	Selleck, USA
Fer-1	S7243	Selleck, USA
Lip-1	S7699	Selleck, USA
LDC7559	GC65393	GLPBIO, China
Chloroquine	S6999	Selleck, USA

Table2: This table lists the primary antibodies and pharmacological agents used in the study, along with their sources and catalog numbers. All reagents were used according to the manufacturer's instructions.
